# Home-Based Exercise Cardiac Telerehabilitation on Adherence and Functional Capacity for Patients After Percutaneous Coronary Intervention in Indonesia: Protocol for a Quasi-Experimental Study

**DOI:** 10.2196/81067

**Published:** 2026-01-16

**Authors:** Wantiyah Wantiyah, Anggoro Budi Hartopo, Sri Setiyarini, Budi Yuli Setianto

**Affiliations:** 1 Faculty of Medicine, Public Health and Nursing, Universitas Gadjah Mada, Yogyakarta, Indonesia; 2 Department of Medical Surgical and Critical Nursing, Faculty of Nursing, Universitas Jember, Jember, Indonesia; 3 Department of Cardiology and Vascular Medicine, Faculty of Medicine, Public Health, and Nursing, Universitas Gadjah Mada, Yogyakarta, Indonesia; 4 Department of Basic and Emergency Nursing, Faculty of Medicine, Public Health and Nursing, Universitas Gadjah Mada, Gedung Ismangoen, Jl. Farmako Sekip, Yogyakarta, 55281, Indonesia, 62 8122701425

**Keywords:** adherence, cardiac rehabilitation, functional capacity, home-based exercise cardiac telerehabilitation, percutaneous coronary intervention

## Abstract

**Background:**

Home-based cardiac rehabilitation is a promising approach to improve access and adherence to cardiac rehabilitation (CR), which remains underutilized. However, monitoring patient adherence and safety during home-based exercise is challenging. Therefore, we develop and implement a home-based exercise cardiac telerehabilitation (HBECTR) program, integrating structured exercise guidance with remote monitoring.

**Objective:**

This trial is designed as a pilot feasibility study to evaluate the practicality, safety, and preliminary effects of HBECTR in patients who have undergone percutaneous coronary intervention (PCI). The primary objective is to analyze the effect of HBECTR on patients’ adherence to CR, while the secondary objective is to measure the effect of HBECTR on the functional capacity of patients after PCI.

**Methods:**

This study is a quasi-experimental nonequivalent control group pretest-posttest. The eligible participants are adults (≥18 y), patients with coronary artery disease after successful PCI, and patients having active health insurance. Patients will be consecutively recruited from Dr. Sardjito General Hospital, Yogyakarta, Indonesia, with a minimum of 60 patients after PCI as samples, divided into 2 groups: an intervention group and a comparison or control group. The intervention group will receive hybrid cardiac rehabilitation. Patients will be scheduled to do exercise twice a week at the hospital and 3 times a week at their home. When patients exercise at home, they will have to wear the smartwatch “CovWatch,” which will be connected to the web-based app (App Rehab Cardio) to monitor their vital signs and the distance of walking. The control group will get usual care (center-based cardiac rehabilitation). The data will be analyzed using the Student *t* test to see if the data comply with the normality assumption. This protocol received approval from the institutional review board of the Faculty of Medicine, Public Health, and Nursing UGM (approval number: KE/FK/0850/EC/2024).

**Results:**

The recruitment of the patients started in August 2024. This study is being funded since February 2025. During the submission, we have recruited 63 patients in both intervention and control groups. The intervention for both groups was finished in June 2025, and the data are being analyzed.

**Conclusions:**

It is expected that the HBECTR program will be feasible and safely implemented in low- and middle-income countries, particularly in Indonesia. By integrating a telerehabilitation model, it is expected that HBECTR will improve patients’ adherence to CR and also increase the functional capacity of patients after PCI.

## Introduction

### Background

Cardiovascular disease (CVD) is the leading cause of death and disability worldwide, with the highest death rates (more than 75%) occurring in low- and middle-income countries [1]. Indonesia reported 765,660 deaths from CVD in 2021, placing Indonesia in the top 20% rank of countries with the highest mortality based on age-standardized mortality from CVD. Furthermore, Indonesia has relatively high rates of non-high-density lipoprotein cholesterol in women and tobacco use in men when compared to other countries globally. Indonesia has implemented 4 of 8 key CVD-related policies. It does have a national action plan for CVDs in place and an operational unit within the Ministry of Health with the responsibility for managing non-communicable diseases; however, the action plan to reduce physical inactivity has not been developed [2].

Cardiac rehabilitation (CR), a multicomplex intervention that is a class 1A recommendation from the American Heart Association and the American College of Cardiology, aims to improve the health condition of patients [3,4], reduce mortality, enhance recovery, and support positive behavioral changes to reduce risk factors [4-7]. Although the effectiveness and benefits of the CR program have been proved, it is still underutilized, and its access is limited [5]. Globally, the level of participation and compliance of patients undergoing CR is still low [8], especially in countries with limited resources [9]. The compliance rate for patients with post-heart attack is 14%‐35% [10] and around 24% for patients with coronary heart disease with various conditions [11]. It means that more than 80% of the patients who are referred to CR do not participate in the program [12]. Patients’ participation and adherence to CR are essential to determine the success of prevention efforts and improve patients’ condition [13].

Exercise-based cardiac rehabilitation (EBCR) is essential for patients undergoing revascularization (postpercutaneous coronary intervention [PCI]) because they often experience decreased exercise capacity and physical activity levels, and both of these indicators are very important for predicting postprocedure morbidity and mortality [14]. Furthermore, these patients also need self-management to improve their health conditions and other clinical outcomes, such as reducing depression and anxiety, mortality, and morbidity and improving their quality of life [15]. EBCR focuses on improving cardiac endurance, exercise capacity, muscle strength, physical activity levels, and quality of life through health education and lifestyle modification [14]. Physical exercise is the main pillar (core stone) of cardiac rehabilitation [13] because it can increase functional capacity and encourage patients to develop and maintain an active lifestyle that can reduce the risk of recurrence in the future [16].

In Indonesia, CR is predominantly implemented in hospital-based or center-based cardiac rehabilitation (CBCR) settings, as the national health insurance scheme—Badan Penyelenggara Jaminan Sosial (BPJS)—currently reimburses only CBCR services. In contrast, home-based cardiac rehabilitation (HBCR), cardiac telerehabilitation, and virtual rehabilitation programs are not yet covered by the insurance system [17]. Although BPJS facilitates access to CBCR, CR services remain limited because not all hospitals in Indonesia have established CR facilities. Similar to other countries in the Southeast Asia region, most available programs are concentrated in tertiary care centers or major urban areas [18]. Dr. Sardjito General Hospital in Yogyakarta is one of the few hospitals in Indonesia that provides CR services and has offered EBCR since 2006. However, the accessibility and continuity of CR remain major challenges, particularly for patients who live far from the hospitals or face transportation and time constraints.

The implementation of EBCR at Dr. Sardjito Hospital also has evolved considerably between the pre- and post-COVID-19 periods, particularly in terms of session frequency and delivery format. Before the pandemic, the EBCR program consisted of 10‐12 sessions, typically conducted 3‐5 times per week. During the pandemic, however, the frequency was reduced to twice per week while maintaining the total number of 10‐12 sessions [17]. At present, most patients participate in CBCR twice weekly for 4 weeks, resulting in approximately 8 sessions overall. The duration and frequency may be extended depending on each patient’s clinical condition or physician’s recommendation. Nevertheless, the current practice remains below the national guideline, which recommends that patients engage in exercise 4‐7 times per week to achieve optimal outcomes [19]. Therefore, incorporating home-based exercise as part of a hybrid or fully HBCR program may serve as a feasible and scalable alternative to meet the recommended exercise frequency, enabling patients to continue rehabilitation safely and effectively at home on nonhospital days.

HBCR has a positive effect on patient self-efficacy [7], which will encourage patients to behave positively. Besides, home-based exercises have been proven to be very effective in improving patient conditions because they can empower patients to monitor the exercises they do independently [13]. Moreover, HBCR has the same safety and effectiveness as hospital or CBCR in improving patient functional capacity and various other clinical outcomes [1,5,20-22]. HBCR has emerged as a promising alternative to CBCR [5,23].

Despite the various advantages and benefits of HBCR, there are several obstacles, such as the absence of clear guidelines regarding dosage and components, its limited implementation in certain cases, and its limited implementation in all countries. The research and implementation of HBCR are mostly in developed countries such as the United States, Australia, and England [5,24].

Adding artificial intelligence that can interpret data and monitor the implementation of CR can increase the safety and feasibility of HBCR. Devices installed or worn by patients are expected to identify the patients’ toleration of exercise dose and monitor the vital signs to ensure patient safety during exercise at home [20]. The use of information technology in its implementation is predicted to increase the long-term effects of HBCR [24]. Smartphones or mobile devices (20/31, 65%), web-based portals (18/31, 58%), and email or SMS (11/31, 35%) are the most often used information technologies to support CR [12]. In this study, we will use a web-based app (dashboard) to support the implementation of home-based exercise cardiac telerehabilitation (HBECTR).

Although several studies support the implementation of telerehabilitation, there are still no definitive data on the efficacy of HBECTR compared to CBCR, thus requiring further research [23,25]. Besides, the biggest challenge today is how to improve patient adherence and compliance in CR, the development of HBCR guidelines, and the implementation of information technology in HBCR that can be applied in countries with limited resources, like Indonesia. Therefore, this study is designed as a pilot feasibility trial to evaluate the practicality, safety, and short-term effects of a digitally supported HBECTR program in Indonesian patients after PCI. This is the first pilot study in Indonesia implementing digitally supported HBCR, particularly using a web-based platform, and addressing a critical gap in low- and middle-income country settings where CR utilization remains low. The pilot nature of this study allows us to examine the feasibility of the program, adherence, safety monitoring, and digital health implementation in a resource-limited setting.

### Objectives

This study aimed to evaluate the effectiveness of an HBECTR program for patients after PCI. The primary objective was to assess the effect of HBECTR on adherence to CR, while the secondary objective was to determine its effect on patients’ functional capacity.

### Hypotheses

In this study, we examined two hypotheses:

1. There is no difference in functional capacity between the HBECTR group and the control group (CG; H_0_) or HBECTR will improve functional capacity compared to the control (H_1_).
2. There is no difference in adherence between the HBECTR group and the CG (H_0_) or HBECTR will improve adherence to CR compared to the control (H_1_).

## Methods

### Study Design

This trial is designed as a pilot feasibility study to evaluate the practicality, safety, and preliminary effects of HBECTR using a quasi-experimental nonequivalent CG pretest-posttest. Participants will be assigned to either the intervention group (IG), which will receive the HBECTR program, or the CG, which will receive standard care (CBCR). Pre- and post-intervention data will be collected to assess the impact of the HBECTR program on patients’ adherence as primary outcomes and their functional capacity as secondary outcomes.

We use a quasi-experimental design with a nonrandomized control group due to the limited number of eligible participants [26], the logistical and resource constraints, and to bringing the implementation closer to the real world. As a pilot study, we want to identify the feasibility of HBECTR in Indonesia. In fact, there is a strong need to further investigate and incorporate synchronous digital CR into the routine of cardiac patients’ secondary prevention routine [27].

### Settings

This study was being conducted since August 2024 and June 2025 at Dr. Sardjito Yogyakarta General Hospital, Indonesia, focusing on patients after PCI. The intervention will be done in the CR center at Dr. Sardjito General Hospital, Yogyakarta, and at the patients’ homes. The research environment will include outpatient clinics and remote monitoring settings where participants will receive either the HBECTR program or standard care. The setting will ensure access to medical records, availability of telehealth technologies, and close coordination with health care professionals to support the implementation and evaluation of the HBECTR program.

### Samples

The population consists of patients after PCI recruited using consecutive sampling. Sample size calculation was performed based on previous studies [15,28], with the minimum difference of 6-minute walk distance as 30 m [29,30]. We determined the patients based on the 95% CI, 80% power, 5% type I error, and 10% dropout rate. A minimum of 60 patients (30 per group) will be included, consistent with recommendations for pilot feasibility studies, which suggest 12‐30 participants per arm to estimate variability and assess feasibility parameters [31,32]. We will carefully select the samples based on the inclusion criteria: (1) patients with coronary artery disease after successful PCI, (2) aged 18 years or above, (3) covered by active health insurance (BPJS), (4) willing to participate and sign informed consent, (5) having internet connectivity at home (for the CG), (6) living with family or having a family member as a caregiver, and (7) able to read and understand Bahasa Indonesia.

The exclusion criteria were as follows: (1) patients at a high cardiovascular risk as defined by the American Association of Cardiovascular and Pulmonary Rehabilitation (2007), the American Heart Association (2001), Société Française de Cardiologie (2002), and Indonesia based on the Indonesia Heart Association (2019), which included any of the following: (a) resting ejection fraction <30%, (b) history of cardiac arrest or sudden death, (c) complex arrhythmia at rest, (d) multiple imputation (MI) or revascularization procedure with complications, (e) congestive heart failure, (f) symptoms and signs of ischemia post-MI or post-revascularization, or (g) clinical depression; (2) inability to walk independently; and (3) having contraindications to participate in CR, such as (a) unstable angina, (b) resting systolic blood pressure (BP) >200 mm Hg or diastolic BP >110 mm Hg (requiring case-by-case evaluation), (c) orthostatic BP drop >20 mm Hg with symptoms, (d) critical aortic valve stenosis, (e) active systemic illness or fever, (f) uncontrolled atrial or ventricular arrhythmia, (g) uncontrolled sinus tachycardia (>120 bpm), (h) uncompensated heart failure, (i) third-degree atrioventricular block without a pacemaker, (j) active pericarditis or myocarditis, (k) new thromboembolic events, (l) ST-segment depression or elevation at rest (>2 mm), (m) uncontrolled diabetes, (n) severe orthopedic conditions preventing physical exercise, and (o) other metabolic issues such as acute thyroiditis, hypokalemia, hyperkalemia, or hypovolemia. Drop-out criteria included (1) patients who only participated once or did not complete the full rehabilitation program (loss to follow-up) and (2) patients who have discontinued the program due to clinical reasons (medical advised).

Both groups will be recruited consecutively using identical eligibility criteria, recruitment procedures, and measurement protocols. This study uses a quasi-experimental nonequivalent control group design where the IG was recruited and completed follow-ups before the initiation of the control or comparison group. Sequential, nonconcurrent enrollment was selected due to practical constraints, including a limited number of eligible patients and complete essential preparatory activities and devices (smartwatch and dashboard setup, system testing, and staff training) prior to delivering the hybrid cardiac telerehabilitation intervention. Similar sequential recruitment approaches are common in pragmatic and feasibility trials where randomization or concurrent enrollment is not operationally feasible [26,33].

However, such nonconcurrent enrollment introduces potential time-related biases that statistical adjustment cannot fully eliminate [33]. To mitigate these concerns, we will implement two strategies. First, in the pre-intervention assessment, we will perform demographic and clinical assessments at baseline across both groups. We will carefully select patients based on the eligibility criteria, standardize all outcome measurements (attendance list, 6MWT) using the same instruments and trained team, and apply identical follow-up schedules. These data will help us statistically adjust for any identified differences, although we recognize that such adjustments may not completely eliminate bias. Second, in propensity score matching (PSM), we will plan to use PSM to equate the groups on key variables measured at baseline, which can help balance characteristics across the groups. Baseline demographic and clinical characteristics will be carefully documented, and differences between the groups will be statistically adjusted using multivariable regression and propensity score methods. The use of PSM enabled the construction of a comparison group comprising individuals similar to the treatment group individuals on most observable characteristics [34].

**Figure 1.**
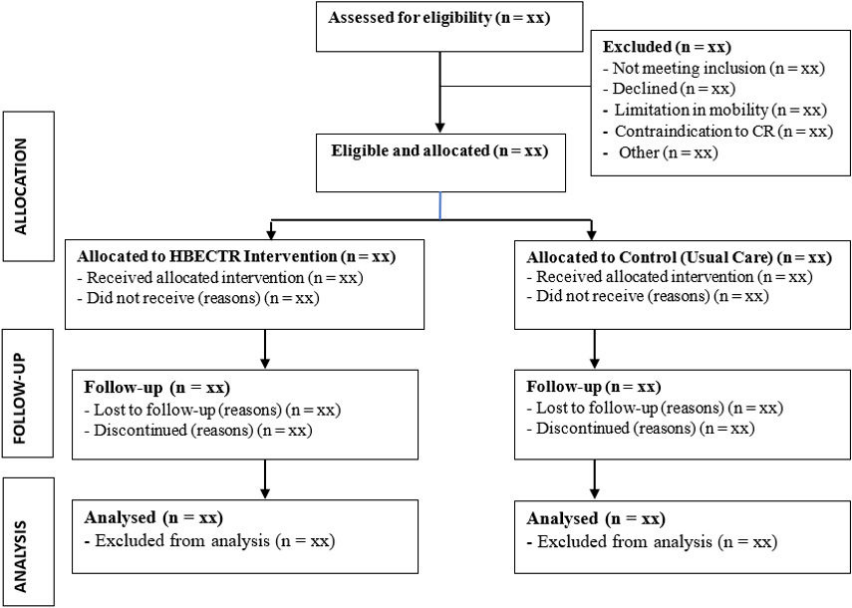
CONSORT (Consolidated Standards of Reporting Trials) flow diagram.

Blinding procedures are often not feasible in quasi-experimental studies due to the absence of randomized assignment and the practical realities of real-world intervention delivery, particularly in exercise, physical activity, and rehabilitation research [35]. In this study, the full blinding of participants and assessors was not possible because of the overt nature of the intervention and logistical constraints. Nevertheless, the risk of bias was mitigated by implementing several strategies: using validated measurement tools; providing objective and standardized outcome assessments; and, wherever feasible, ensuring that data analysts were blinded to group allocation during the data analysis phase. Data were coded such that the analysts were unaware of group assignments until after the completion of initial analysis (see Figure 1).

### Study Procedures

To assess the efficacy of the HBECTR program, we divided subjects into 2 groups: the intervention group and the control group (Table 1). The CG will receive standard hospital CR or CBCR, while the treatment group will receive HBECTR. We define HBECTR as exercise-based cardiac rehabilitation conducted in a hybrid setting, both in the hospital and at the patient’s home, supported by a telemonitoring system.

We will use a telemonitoring system consisting of a smartwatch (CovWatch) produced by PT CovWatch Karya Nusantara, Indonesia, connected via the internet to the web-based app (dashboard), namely “Telerehabilitasi Kardiovaskular (TEKAD)” (App Rehab Cardio) [36]. We developed and conducted usability testing before the dashboard was released (will be reported separately). Prior to the initiation of the study, an accuracy test will be conducted on 10‐20 patients as part of the CovWatch protocol. A standardized calibration check will be performed at baseline by comparing CovWatch-derived heart rate (HR) with a clinical pulse oximeter at rest and during walking test. Data validity will be monitored through automated system flags for missing data, connectivity loss, and out-of-range physiological values. Mitigation strategies will include repeating measurements, instructing participants on proper device placement (detailed in the guidance book for patient), verifying battery status, and resynchronizing data in case of delayed transmission. All sensor data will be stored with time stamps to identify anomalies and enable manual quality checks.

Before starting the exercise, all patients will undergo a pretest to measure functional capacity using the 6-minute walk test (6MWT) [37]. This pretest is carried out when the patient is still hospitalized or during the first check-up after PCI, according to hospital policy. The cardiologist will prescribe an exercise regime tailored by patients based on this baseline 6MWT, in accordance with the CR guidelines in Indonesia [19]. After the patients have finished all CR programs, they will do the 6MWT for a posttest. The 6MWT for both pretest and posttest will be done at the CR center, Dr. Sardjito General Hospital, Yogyakarta. The intervention of this study will be reported according to the TIDieR (Template for Intervention Description and Replication) [38], in conjunction with the CERT (Consensus on Exercise Reporting Template) [39] and mERA (mHealth Evidence Reporting and Assessment; Checklist1^4^).

**Table 1.** Exercise prescription based on frequency, intensity, time, and type (frequency, intensity, time, and type) for the control group and the intervention group.

Dose	CG^a^ (CBCR^b^)	IG^c^ (HBECTR^d^)
Frequency	Standard care included 2 sessions per week at the hospital	5 sessions per week, divided into 2 sessions per week at the hospital and 3 sessions per week at home
Intensity	Moderate	Moderate
Time	45 min Warm-up: 5 min Core-exercise: 30 min Cool-down: 10 min	45 min Warm-up: 5 min Core-exercise: 30 min Cool-down: 10 min
Type	Aerobic (ergo cycle and treadmill)	Aerobic: ergo cycle and treadmill at the hospital; or walking on flat ground at home
Monitoring	Hospital: supervised by CR^e^ nurse based on vital signs, distance, RPE^f^, and %HRR^g^	Hospital: supervised by CR nurse based on HR^h^, BP^i^, SaO_2_^j^, distance, RPE, and %HRR Home: monitored by smartwatch connected to dashboard (App Rehab Cardio), based on HR, BP, SaO_2_, distance, and patients' complaints

a CG: control group.

b CBCR: center-based cardiac rehabilitation.

c IG: intervention group.

d HBECTR: home-based exercise cardiac telerehabilitation.

e CR: cardiac rehabilitation.

f RPE: rate of perceived exertion

g HRR: heart rate reserve.

h HR: heart rate.

i BP: blood pressure.

j SaO_2_: oxygen saturation.

### The Intervention Group

The patients in the treatment group will be prescribed an individually EBCR program at home and in the hospital. The targeted heart rate and walking distance targets will be set individually based on baseline 6MWT. The intensity progression will be evaluated weekly if tolerated. Exercise education and an exercise guidance book will be provided to patients before the program begins. In the hospital, patients do a 45-minute exercise session consisting of a warm-up (5 min), core exercise (30 min) in the form of walking on a treadmill, and cool down (10 min). This exercise is carried out twice a week over 4 weeks in the hospital (a total of 8 sessions) and is directly supervised by a certified cardiac rehab nurse (Table 1).

**Figure 2.**
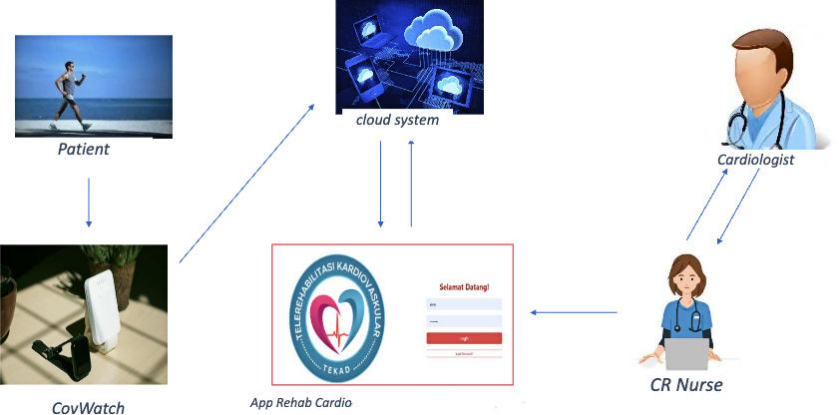
Real-time monitoring in home-based exercise cardiac telerehabilitation (HBECTR). CR: cardiac rehabilitation. App Rehab Cardio can be accessed via the web [36].

During exercise at home, subjects will be monitored by a “TEKAD” telemonitoring system. Patients will wear CovWatch that will be connected via the internet to a web-based system (App Rehab Cardio). Prior to the exercise at home, the researcher and the IT team will ensure that the CovWatch is connected to the dashboard App Rehab Cardio to facilitate the real-time monitoring of patients’ vital signs during exercise (see Figure 2). Home exercise will be carried out for 45 minutes, consisting of 5 minutes of warm-up (eg, simple stretching or slow walking), 30 minutes of core exercise walking based on the exercise dose and patient’s ability, and 10 minutes of cooling down. Vital signs, such as pulse, BP, and oxygen saturation, will be monitored via CovWatch and displayed on the dashboard. Home exercises will be prescribed 3 days a week over 4 weeks (12 sessions in total), with a schedule adjusted by the researchers so as not to coincide with the hospital exercise sessions. Patients will be evaluated and receive weekly exercise reminders directly when they perform exercise at the hospital.

### Real-Time Monitoring

During exercise at home, subjects will be monitored by a nurse remotely through telemonitoring (TEKAD). With high-precision sensors, the smartwatch “CovWatch” will continuously track the patient’s HR, BP, oxygen saturation level during exercise, and estimated walking distance. The system is connected via the internet to the dashboard (App Rehab Cardio; see Figure 2).

### The Control Group

The CG will be enrolled in a cardiac rehabilitation program in the hospital. Patients in this group also will receive exercise education before starting the program. They will be scheduled for 2 sessions per week or 8 sessions in total to do individual exercise at the hospital under a CR nurse’s supervision. Based on hospital policy, patients are also suggested to exercise at home independently (without monitoring). Hospital exercises consist of a 5-minute warm-up, 30-minute core exercise (walking on a treadmill), and a 10-minute cool-down (Table 1).

### Measurement

In this study, respondents will be asked to fill out the participant characteristics, including age, sex, BMI, and medical history, which were collected at baseline through direct interviews and medical record reviews. Sociodemographic information, such as marital status, educational level, occupation, insurance ownership, and average monthly income, was also gathered. In addition, participants were asked about their smoking history (categorized as current, former, or never smoked) and any past or current illnesses, diseases, or risk factors. Clinical information was obtained, including the patient’s medical diagnosis, ejection fraction, prescribed therapies, weight and height, as well as the schedule of heart polyclinic control and cardiac rehabilitation, if applicable.

Adherence to the exercise-based cardiac rehabilitation program as the primary outcome will be measured by recording the number of sessions attended by each participant. Based on a previous study [40], adherence will be calculated as the number of attended sessions divided by the total number of prescribed sessions, expressed as a percentage, with a minimum of 80% session completion considered adherent. Attendance during in-hospital exercise sessions will be directly monitored by CR nurses and recorded in the CR attendance form, while home-based exercise attendance for IG will be verified through the web-based dashboard system. Adherence will be monitored by smartwatch logs and dashboard analytics. We will apply this comprehensive approach to ensure accurate measurement of each participant’s adherence to the prescribed rehabilitation program.

Functional capacity will be assessed using the 6MWT to measure the total distance (in meters) during 6 minutes test (6-minute walk distance) [19,37]. In addition, physiological data such as HR, BP, and oxygen saturation will be continuously monitored using the telemonitoring system (see Figure 2), ensuring the safety and accuracy of HBECTR. To provide a more comprehensive assessment of functional improvement, peak oxygen uptake (VO_₂_ peak) or metabolic equivalents will be estimated based on the distance covered in the 6MWT, using established predictive equations [41,42]. These measurements collectively will provide robust data on the participants’ functional performance following the intervention.

 The primary and secondary outcomes will be measured after participants have finished the intervention (4-wk follow-up). We selected a 4-week follow-up because this is a pilot feasibility study designed to assess the early feasibility, safety, and adherence of HBECTR. Short-duration assessments are well established and appropriate for pilot and feasibility trials, particularly in digital and cardiac telerehabilitation research [43,44]. Besides, the guideline of CR in Indonesia based on the Indonesia Heart Association also recommends conducting an evaluation after 1 month (4 wk) of phase II CR [19]. Furthermore, early improvements in functional capacity or cardiopulmonary endurance often occur within the first month [5,45], making a 4-week evaluation appropriate for assessing initial signal of effect.

We consider extending the follow-up (3‐6 mo follow-up) for our next trial to ensure the sustainability and efficacy of this program. In addition to feasibility and functional outcomes, we will record hospital readmissions during intervention, and patients will be followed up for 3‐6 months. The ability of patients to do their daily activities will be monitored and recorded using a self-report.

In addition, the implementation outcomes will be assessed using the RE-AIM (Reach, Effectiveness, Adoption, Implementation, and Maintenance) framework [46]. We will report recruitment and representativeness (Reach), adherence and safety (Effectiveness), provider uptake (Adoption), fidelity and dose (Implementation), and short-term intention to continue (Maintenance). Qualitative interviews will be added to explore participants’ experiences and barriers during intervention. Furthermore, we will calculate cost-effectiveness by determining the cost per patient, application development, wearable device production, and internet provision.

### Statistical Analysis Planning

All data will be analyzed using the IBM SPSS software (version 29). We will use MI for missing data. MI is a widely used technique for handling missing data in various fields, including medical, social, and ecological research.

Data analysis will be performed primarily on participants who complete both baseline and postintervention assessments, regardless of their adherence level during the intervention period (*completer analysis*). Participants who withdraw or are lost to follow-up before the posttest will be excluded from outcome analysis but will be included in the descriptive analysis of recruitment, adherence, and retention rates. This approach is considered appropriate for pilot quasi-experimental studies, where the primary aim is to assess feasibility, acceptability, and signal of effect rather than to test definitive efficacy [31,35].

To minimize potential bias due to nonrandom assignment, baseline characteristics between the groups will be compared using 2-tailed independent *t* tests or chi-square tests. In addition, propensity score adjustment will be applied in exploratory models to control for potential confounding variables (eg, age, sex, baseline functional capacity, comorbidity), following recommendations for quasi-experimental design analysis [33].

All statistical analyses and reporting will follow the principles outlined in the CONSORT-EHEALTH 2011 (Consolidated Standards of Reporting Trials of Electronic and Mobile Health Applications and Online Telehealth) [47] (Checklist 3). Descriptive statistics will summarize feasibility outcomes (recruitment, adherence, completion rates, safety events), while inferential tests (2-tailed paired *t* tests or analysis of covariance) will be used to explore within- and between-group changes (including means and SDs) in functional capacity and adherence measures. Statistical significance will be interpreted cautiously, given the pilot nature and limited sample size of the study [31,35].

To analyze the effects of HBECTR on patient adherence as the primary outcome and functional capacity as the secondary outcome, there is no formal multiplicity adjustment planned. The data analysis based on normality assumption will be tested using the Shapiro-Wilk test. If the data comply with the normal assumption, we will use the Student *t* test, and vice versa, nonparametric tests will be used if assumptions are violated. In all statistical tests, *P*<.05 (95% CI) will be considered statistically significant. This structured approach will ensure that statistical methods are properly tailored to the data, supporting robust and meaningful interpretation of the results. The statistical analysis plan has been registered within the protocol to the institutional ethics committee board with the approval number KE/FK/0850/EC/2024.

### Safety and Monitoring

Patient safety during exercise is crucial and must be taken into consideration. Clinical supervision and efforts to minimize complications during the exercise program must be implemented to ensure the safety of CR [19]. In this study, the efforts to supervise and maintain patient safety during exercise will involve several aspects related to the program and patients. All patients must obtain a referral from a cardiologist and will undergo an assessment before, during, and after the program. During the recruitment of potential subjects, screening will be conducted, and only patients with a low and moderate risk will be included. We will also educate patients about the signs and symptoms of cardiovascular emergencies, both during exercise at the hospital and home, such as chest pain, irregular heartbeat, weight gain, and shortness of breath. A guidebook will be provided to patients, which details how to perform exercises safely at home and how to use the smartwatch for monitoring. The guidebook also includes the WhatsApp numbers of the researchers and cardiac rehab team and emergency contact details.

Regarding the program, risk stratification screening will be conducted prior to exercise, and patients will be supervised and monitored during exercise. Advances in technology have enabled the remote monitoring of patients, especially when exercise is performed at home or outside the hospital. The safety of remotely delivered CR has been demonstrated by the low long-term rates of reported exercise−related adverse events (AEs), serious adverse events (SAEs), and rehospitalization incidences [48]. Hence, this study will entail the implementation of a telemonitoring system, utilizing the “TEKAD” dashboard for monitoring patients’ vital signs during exercise to ensure the safety of the program (Figure 2).

Prior to participation, all patients will receive comprehensive explanations of the program’s procedures, safety protocols, and communication flow, which will be reinforced again at the start of the intervention. Participants will receive detailed instructions on the prescribed exercise regimen to be performed both at the hospital and at home. They will be guided on the appropriate type, intensity, and duration of exercise and will be encouraged to report any symptoms or discomfort experienced during exercise. If patients experience any adverse symptoms, such as chest pain, shortness of breath, unusual fatigue, or other discomfort, they will be instructed to immediately stop exercising and rest.

During exercise at home, patients will be able to contact the cardiac rehabilitation team via WhatsApp in case of any complaints or concerns. Conversely, if the team detects abnormal changes in vital signs (eg, HR, BP, or oxygen saturation) during remote monitoring, they will proactively contact the patient to inquire about their condition. Then, if necessary, patients will be advised to stop the exercise and take adequate rest.

Furthermore, the risk of AEs and SAEs in EBCR is low. However, it is increased during vigorous intensity exercises relative to rest [49] and intensive exercise training (≥7 times per week) [50]. Therefore, this study will implement the moderate intensity exercise within 5 times per week. Besides, the exercise prescription will be individualized based on the 6MWT baseline, following the CR guideline in Indonesia [19]. All AEs or SAEs during the study will be reported to the cardiologist who will be supervising this program. These also will be documented in the case report form and reported to the ethical board.

### Ethical Considerations

This study protocol adhered to the Declaration of Helsinki and received approval from the institutional review board of the Faculty of Medicine, Public Health, and Nursing, Universitas Gadjah Mada (approval number KE/FK/0850/EC/2024). All participants signed an informed consent form before the study commenced, ensuring their voluntary participation and the protection of their confidentiality throughout the study. The subjects in both groups who will attend on-site rehabilitation sessions at the hospital will receive financial compensation to reimburse transportation costs. This compensation will be provided to support the travel costs incurred during study participation and will not intend to constitute an inducement to participate. The amount and mechanism of reimbursement have been reviewed and approved by the institutional ethics committee.

Emphasizing that the health information of patients is sensitive and the leakage of the information could bring both ethical and economical risks [51], all data obtained will be carefully documented and stored in an encrypted system, and patient confidentiality will be maintained following research ethics standards. Furthermore, the app or dashboard is available only to the researcher team and is password-protected.

This trial was retrospectively registered to the ANZCTR with registry ID: ACTRN12625001400459 (IRRIDDERR1-10.2196/81067). The first enrolment was started at August 14, 2024, while the trial registration was submitted on October 15, 2025 The trial was registered retrospectively due to an administrative and tehnical problem related to the app development.

## Results

The recruitment of the subject started from August 2024. This study is being funded since February 2025. During the submission, we have recruited 63 subjects for both IG and CG. The intervention for both groups finished in June 2025, and the data are being analyzed. It is expected that the data analysis will be finished in November 2025 and that the results will be published in December 2025.

## Discussion

The low adherence of patients to participate in CR programs, particularly after PCI, remains a significant challenge in Indonesia and other countries. Despite class 1 recommendations for CR as part of secondary prevention, CBCR is often underutilized due to various factors, including transportation difficulties, limited availability, and low patient motivation. This study aims to address these challenges by implementing an HBECTR program to enhance patients’ adherence to CR.

This HBECTR intervention protocol offers an innovative approach to CR by leveraging technology to provide continuous monitoring, guidance, and feedback to patients. Previous studies have indicated that home-based cardiac telerehabilitation can be as effective as traditional hospital−based programs in improving functional capacity, adherence, and quality of life [15]. However, there has been limited evidence on home-based cardiac telerehabilitation implementation in Indonesia, particularly in post-PCI patients.

By integrating a telerehabilitation model in HBCR, this study may address several barriers to traditional CR, including geographical challenges and logistical issues. Digital technologies have revolutionized CR, offering flexible and novel approaches to care. The integration of digital health technologies and artificial intelligence in remote CR is transforming traditional paradigms, providing real-time access to health data, and enhancing patient self-management. Mobile and digital CR models, including synchronous or real-time digital interventions, are addressing accessibility barriers and promoting equity in health care delivery [23,27].

Despite the benefit or positive aspect of HBECTR, there are several challenges such as digital literacy, data privacy, and security that must be addressed to ensure the inclusive implementation of CR. Moreover, the shift toward digital CR raises concerns about cost, safety, and potential depersonalization of therapeutic relationships [27].

Furthermore, several potential limitations should be acknowledged in this quasi-experimental study. First, the intervention may yield null or minimal effects, particularly on adherence to cardiac rehabilitation and functional capacity, due to variability in participant engagement, baseline fitness levels, or external barriers such as comorbidities or limited motivation. Second, feasibility challenges are anticipated, including variable access to technology, differences in digital literacy, difficulties in remotely monitoring safety, and potential missing data from nonadherence or technical issues. Using a wearable device (smartwatch) is preferable to most patients; however, it has challenges in its implementation and needs further exploration to gain a deeper understanding of patients’ needs and preferences [52]. Finally, resource limitations may constrain implementation, including limited personnel for supervision, insufficient devices for remote monitoring, costs associated with web-based platforms, and the need for staff training and scheduling coordination.

In addition, this study also has limitations, including its modest sample size, and the short follow-up duration limits its generalizability to the broader population. These are intentional features of a pilot feasibility trial that focuses on testing feasibility, safety, and preliminary efficacy signals rather than providing conclusive evidence of effectiveness [32]. Clinical trials of physical activity and rehabilitation interventions are challenging; therefore, researchers can use pilot and feasibility studies to enhance the rigor of future trials [35].

Despite these challenges, the study provides valuable insights into the practicality and potential impact of HBECTR, informing the design of larger, fully powered trials. Future research is needed to enhance the study in multicenters with multiple outcomes. Longer-term effects, sustainability, and cost-effectiveness should be evaluated in a subsequent definitive trial.

## Supplementary material

10.2196/81067 Checklist 1 TIDIeR (Template for Intervention Description and Replication) checklist.

10.2196/81067 Checklist 2 CERT (Consensus on Exercise Reporting Template) checklist.

10.2196/81067 Checklist 3 CONSORT-EHEALTH (Consolidated Standards of Reporting Trials of Electronic and Mobile Health Applications and Online TeleHealth) checklist.

10.2196/81067 Checklist 4 mERA (mobile health [mHealth] Evidence Reporting and Assessment) checklist.

## Abbreviations

6MWT: 6-minute walk test
AE: adverse event
BP: blood pressure
BPJS: Badan Penyelenggara Jaminan Sosial
CBCR: center-based cardiac rehabilitation
CERT: Consensus on Exercise Reporting Template
CG: control group
CONSORT: Consolidated Standards of Reporting Trials
CONSORT-EHEALTH: Consolidated Standards of Reporting Trials of Electronic and Mobile Health Applications and Online Telehealth
CR: cardiac rehabilitation
CVD: cardiovascular disease
EBCR: exercise-based cardiac rehabilitation
HBCR: home-based cardiac rehabilitation
HBECTR: home-based exercise cardiac telerehabilitation
HR: heart rate
IG: intervention group
mERA: mHealth Evidence Reporting and Assessment
MI: multiple imputation
PCI: percutaneous coronary intervention
PSM: propensity score matching
RE-AIM: Reach, Effectiveness, Adoption, Implementation, and Maintenance
SAE: severe adverse event
TEKAD: Telerehabilitasi Kardiovaskular
TIDieR: Template for Intervention Description and Replication

## References

[1] (2025). Cardiovascular diseases (CVDs). World Health Organization.

[2] (2025). Cardiovascular insight for Indonesia. World Heart Observatory.

[3] Woodgate J, Brawley LR (2008). Self-efficacy for exercise in cardiac rehabilitation: review and recommendations. J Health Psychol.

[4] Simon M, Korn K, Cho L, Blackburn GG, Raymond C (2018). Cardiac rehabilitation: a class 1 recommendation. Cleve Clin J Med.

[5] Thomas RJ, Beatty AL, Beckie TM (2019). Home-based cardiac rehabilitation: a scientific statement from the American Association of Cardiovascular and Pulmonary Rehabilitation, the American Heart Association, and the American College of Cardiology. J Am Coll Cardiol.

[6] Chindhy S, Taub PR, Lavie CJ, Shen J (2020). Current challenges in cardiac rehabilitation: strategies to overcome social factors and attendance barriers. Expert Rev Cardiovasc Ther.

[7] Poortaghi S, Baghernia A, Golzari SEJ, Safayian A, Atri SB (2013). The effect of home-based cardiac rehabilitation program on self efficacy of patients referred to cardiac rehabilitation center. BMC Res Notes.

[8] Ritchey MD, Maresh S, McNeely J (2020). Tracking cardiac rehabilitation participation and completion among Medicare beneficiaries to inform the efforts of a national initiative. Circ Cardiovasc Qual Outcomes.

[9] Lima AP, Nascimento IO, Oliveira ACA, Martins THS, Pereira DAG, Britto RR (2019). Home-based cardiac rehabilitation in Brazil’s public health care: protocol for a randomized controlled trial. JMIR Res Protoc.

[10] Bakhshayeh S, Sarbaz M, Kimiafar K, Vakilian F, Eslami S (2021). Barriers to participation in center-based cardiac rehabilitation programs and patients’ attitude toward home-based cardiac rehabilitation programs. Physiother Theory Pract.

[11] Beatty AL, Brown TM, Corbett M (2021). Million Hearts cardiac rehabilitation think tank: accelerating new care models. Circ Cardiovasc Qual Outcomes.

[12] Wongvibulsin S, Habeos EE, Huynh PP (2021). Digital health interventions for cardiac rehabilitation: systematic literature review. J Med Internet Res.

[13] Chatzitofis A, Monaghan D, Mitchell E (2015). HeartHealth: a cardiovascular disease home-based rehabilitation system. Procedia Comput Sci.

[14] Prabhu NV, Maiya AG, Prabhu NS (2020). Impact of cardiac rehabilitation on functional capacity and physical activity after coronary revascularization: a scientific review. Cardiol Res Pract.

[15] Zhong W, Fu C, Xu L (2023). Effects of home-based cardiac telerehabilitation programs in patients undergoing percutaneous coronary intervention: a systematic review and meta-analysis. BMC Cardiovasc Disord.

[16] Achttien RJ, Staal JB, van der Voort S (2013). Exercise-based cardiac rehabilitation in patients with coronary heart disease: a practice guideline. Neth Heart J.

[17] Hartopo AB, Arso IA, Ambari AM, Dwiputra B, Radi B (2022). Exercise-based cardiac rehabilitation adaptation protocol during Covid-19 pandemic achieved similar results as compared to non-pandemic usual practice: a single center experience. J Med Sci.

[18] Chowdhury M, Heald FA, Turk-Adawi K (2021). Availability and delivery of cardiac rehabilitation in South-East Asia: How does it compare globally?. WHO South East Asia J Public Health.

[19] Radi B, Tiksnadi BB, Dwiputra B, Sarvasti D, Ambari AM (2019). Panduan rehabilitasi kardiovaskular (guidelines for cardiovascular rehabilitation). https://www.inaheart.org/wp-content/uploads/2025/10/Panduan-Rehabilitasi-Kardiovaskular.pdf.

[20] Stefanakis M, Batalik L, Antoniou V, Pepera G (2022). Safety of home-based cardiac rehabilitation: a systematic review. Heart Lung.

[21] Anderson L, Sharp GA, Norton RJ (2017). Home-based versus centre-based cardiac rehabilitation. Cochrane Database Syst Rev.

[22] Schopfer DW, Whooley MA, Allsup K (2020). Effects of home-based cardiac rehabilitation on time to enrollment and functional status in patients with ischemic heart disease. J Am Heart Assoc.

[23] Zhang S, Wang Y, Wu J, Ma C, Meng X (2025). Effectiveness of smartwatch device on adherence to home-based cardiac rehabilitation in patients with coronary heart disease: randomized controlled trial. JMIR Mhealth Uhealth.

[24] Liu J, Wang L, Fang H, Wang X, Wu L, Zhang J (2022). Home-based cardiac rehabilitation: a review of bibliometric studies and visual analysis of CiteSpace (2012–2021). Medicine (Abingdon).

[25] Antoniou V, Davos CH, Kapreli E, Batalik L, Panagiotakos DB, Pepera G (2022). Effectiveness of home-based cardiac rehabilitation, using wearable sensors, as a multicomponent, cutting-edge intervention: a systematic review and meta-analysis. J Clin Med.

[26] Harris AD, McGregor JC, Perencevich EN (2006). The use and interpretation of quasi-experimental studies in medical informatics. J Am Med Inform Assoc.

[27] Pepera G, Antoniou V, Su JJ, Lin R, Batalik L (2024). Comprehensive and personalized approach is a critical area for developing remote cardiac rehabilitation programs. World J Clin Cases.

[28] Grace SL, Midence L, Oh P (2016). Cardiac rehabilitation program adherence and functional capacity among women: a randomized controlled trial. Mayo Clin Proc.

[29] Gremeaux V, Troisgros O, Benaïm S (2011). Determining the minimal clinically important difference for the six-minute walk test and the 200-meter fast-walk test during cardiac rehabilitation program in coronary artery disease patients after acute coronary syndrome. Arch Phys Med Rehabil.

[30] Keteyian SJ, Grimshaw C, Ehrman JK (2024). The iATTEND trial: a trial comparing hybrid versus standard cardiac rehabilitation. Am J Cardiol.

[31] Julious SA (2005). Sample size of 12 per group rule of thumb for a pilot study. Pharm Stat.

[32] Thabane L, Ma J, Chu R (2010). A tutorial on pilot studies: the what, why and how. BMC Med Res Methodol.

[33] Tilquin C (1976). Patient classification does work. Dimens Health Serv.

[34] White H, Sabarwal S (2014). Quasi-experimental design and methods. https://www.betterevaluation.org/sites/default/files/Quasi-Experimental_Design_and_Methods_ENG.pdf.

[35] El-Kotob R, Giangregorio LM (2018). Pilot and feasibility studies in exercise, physical activity, or rehabilitation research. Pilot Feasibility Stud.

[36] App rehab cardio. https://tekad.covwatch.net/.

[37] Arena R, Myers J, Williams MA (2007). Assessment of functional capacity in clinical and research settings: a scientific statement from the American Heart Association Committee on Exercise, Rehabilitation, and Prevention of the Council on Clinical Cardiology and the Council on Cardiovascular Nursing. Circulation.

[38] Hoffmann TC, Glasziou PP, Boutron I (2014). Better reporting of interventions: template for intervention description and replication (TIDieR) checklist and guide. BMJ.

[39] Slade SC, Dionne CE, Underwood M, Buchbinder R (2016). Consensus on Exercise Reporting Template (CERT): explanation and elaboration statement. Br J Sports Med.

[40] McGregor G, Powell R, Begg B (2023). High-intensity interval training in cardiac rehabilitation: a multi-centre randomized controlled trial. Eur J Prev Cardiol.

[41] Cahalin LP, Mathier MA, Semigran MJ, Dec GW, DiSalvo TG (1996). The six-minute walk test predicts peak oxygen uptake and survival in patients with advanced heart failure. Chest.

[42] Tiksnadi BB, Ambari AM, Adriana M (2019). Uji Jalan 6 Menit (UJ6M) pada Pasien Pasca Sindrom Koroner Akut (Six-minute walk test in patients after acute coronary syndrome). Indones J Cardiol.

[43] Mastorci F, Lazzeri MFL, Ait-Ali L (2025). Home-based intervention tool for cardiac telerehabilitation: protocol for a controlled trial. JMIR Res Protoc.

[44] Varnfield M, Karunanithi M, Lee CK (2014). Smartphone-based home care model improved use of cardiac rehabilitation in postmyocardial infarction patients: results from a randomised controlled trial. Heart.

[45] Zhang L, Ge Y, Zhao W (2025). A 4-week mobile app-based telerehabilitation program vs conventional in-person rehabilitation in older adults with sarcopenia: randomized controlled trial. J Med Internet Res.

[46] Holtrop JS, Estabrooks PA, Gaglio B (2021). Understanding and applying the RE-AIM framework: clarifications and resources. J Clin Transl Sci.

[47] Eysenbach G, CONSORT-EHEALTH Group (2011). CONSORT-EHEALTH: improving and standardizing evaluation reports of Web-based and mobile health interventions. J Med Internet Res.

[48] Antoniou V, Kapreli E, Davos CH, Batalik L, Pepera G (2024). Safety and long-term outcomes of remote cardiac rehabilitation in coronary heart disease patients: a systematic review. Digit Health.

[49] Guy JM, Wilson M, Schnell F (2019). Incidence of major adverse cardiac events in men wishing to continue competitive sport following percutaneous coronary intervention. Arch Cardiovasc Dis.

[50] Mons U, Hahmann H, Brenner H (2014). A reverse J-shaped association of leisure time physical activity with prognosis in patients with stable coronary heart disease: evidence from a large cohort with repeated measurements. Heart.

[51] Su JJ, Chan MHS, Ghisi G de M (2025). Real-world mobile health implementation and patient safety: multicenter qualitative study. J Med Internet Res.

[52] Sarvestan J, Baker KF, Del Din S (2024). Exploring the effect of sampling frequency on real-world mobility, sedentary behaviour, physical activity and sleep outcomes measured with wearable devices in rheumatoid arthritis: feasibility, usability and practical considerations. Bioengineering (Basel).

