# Supplementary material for: Home-Based Exercise Cardiac Telerehabilitation on Adherence and Functional Capacity for Patients After Percutaneous Coronary Intervention in Indonesia: Protocol for a Quasi-Experimental Study
Source: JMIR Res Protoc. 2026 Jan 16;15:e81067. doi: 10.2196/81067 (PMC12810742; doi:10.2196/81067)
Supplement: Checklist 1 [file resprot-v15-e81067-s001.pdf]

TIDieR

HBECTR (Home-based Exercise Cardiac Telerehabilitation) study

In this study, we will use web-based application (dashboard) to support the implementation of home-based exercise cardiac Telerehabilitation (HBECTR).

Why:

To improve adherence and functional capacity in post-PCI patients by overcoming barriers to centre-based CR access in Indonesia

INTRODUCTION

Although many support the implementation of telerehabilitation, there is still no definitive data on the efficacy of HBECTR compared to CBCR, so it still requires further research [25], [28]. Besides, the biggest challenge today is how to improve patient adherence and compliance in CR, the development of HBCR guidelines, and the implementation of information technology in HBCR that can be applied in countries with limited resources, like Indonesia. Therefore, this study is designed as a pilot feasibility trial to evaluate the practicality, safety, and short-term effects of a digitally supported home-based exercise cardiac telerehabilitation (HBECTR) program in Indonesian post-PCI patients. This is the first pilot study in Indonesia implementing digitally supported HBCR, particularly using web-based platform, addressing a critical gap in LMIC settings where CR utilization remains low. The pilot nature of this study allows us to examine the feasibility of the program, adherence, safety monitoring, and digital health implementation in a resource-limited setting.

OBJECTIVES

This study aimed to evaluate the effectiveness of a home-based exercise cardiac telerehabilitation (HBECTR) program for patients after percutaneous coronary intervention (PCI). The primary objective was to assess the effect of HBECTR on adherence to cardiac rehabilitation (CR), while the secondary objective was to determine its effect on patients' functional capacity.

What (material):

Smartwatch connected to web-based application (dashboard "TEKAD", educational booklet, exercise logbook

(1) The Intervention Group (IG)

Subjects in the treatment group will be prescribed an individually EBCR program at home and in the hospital. The heart rate (HR) and walking distance targets individualized based on baseline 6MWT; and the intensity progression weekly if tolerated. Exercise education and exercise guidance book will be provided before the program begins. During the exercise in the hospital, subjects do a 45-minute exercise session consisting of a warm-up (5 minutes), core exercise (30 minutes) in the form of walking on a treadmill, and cool down (10 minutes). This exercise is carried out twice a week over four weeks in the hospital (a total of 8 sessions) and is directly supervised by a certified cardiac rehab nurse (Table 2).

During exercise at home, subjects will be monitored by a telemonitoring system namely "*Telerehabilitasi Kardiovaskular*" or TEKAD. Patients wear a smart watch (CovWatch) that will be connected to a web-based system (http://tekad.covwatch.net). Before starting the exercise at home, the researcher and the IT team will ensure that the CovWatch is connected to the dashboard (App Rehab Cardio) to ensure the real-time monitoring of patient's vital sign during exercise (see figure 2). Home exercise is carried out for 45 minutes consisting of a 5-minute warm-up (e.g. simple stretching or slow walking), 30 minutes of core exercise walking based on the exercise dose and patient's ability, and 10 minutes of cooling down. Vital signs such as pulse, blood pressure, and oxygen saturation were monitored via smartwatch and displayed on the dashboard. Home exercises were performed 3 days a week over 4 weeks (12 sessions in total), with a schedule adjusted by the researchers so as not to coincide with the hospital exercise sessions. Subjects will be evaluated and receive weekly exercise reminders directly when they do exercise at hospital.

Real-time Monitoring

During exercise at home, subjects will be monitored by a nurse remotely trough a telemonitoring (TEKAD). With high-precision sensors, the smartwatch "covwatch" will continuously tracks the patient's heart rate, blood pressure, oxygen saturation level during exercise, and estimated walking distance. The system is connected via internet to the dashboard (App Rehab Cardio) (see figure 2).

What (procedures):

(a) 2 supervised hospital sessions/week for 4 weeks;
(b) 3 home-based sessions/week monitored in real-time by smartwatch connected to web-based application system;
(c) telemonitoring and weekly feedback

Study Procedures

To assess the efficacy of the HBECTR program, we divided subjects into two groups: the intervention group (IG) and the control group (CG) (Table 2). The control group will receive standard hospital or center-based CR (CBCR), while the treatment group will receive Home-based exercise cardiac telerehabilitation (HBECTR). We define HBECTR as an exercise-based cardiac rehabilitation conducted in a hybrid setting, both in the hospital and at the patient's home, supported by a telemonitoring system. During exercise at the hospital, subjects from both groups will be supervised directly by CR nurses. Meanwhile, when the subjects of intervention group doing exercise at home, they will be monitored through a telemonitoring system using web-based application (dashboard) that has been developed by the researcher. The researcher team developed and conducted usability testing before the dashboard released (reported separately). During exercise at home, the subject will wear a smart watch (Covwatch) produced by PT Covwatch Karya Nusantara (PT CKN) Indonesia, which record the patient's vital signs and estimated walking distance. The system is connected via internet to the dashboard at the hospital (App Rehab Cardio). During exercise, the subject is also expected to be accompanied by family.

Before starting the exercise, all subjects will undergo a pre-test to measure functional capacity using the 6-Minute Walk Test (6MWT) [37]. This pre-test is carried out when the patient is still hospitalized or during the first check-up after PCI, according to hospital policy. The Cardiologist will prescribe an exercise regime tailored by patients based on this baseline 6MWT, in accordance with the CR guidelines in Indonesia [21]. After the subjects finished all CR program, they will do the 6mwt for post-test. The 6mwt for both pre-test and post-test will be done at CR center, Dr. Sardjito General Hospital, Yogyakarta.

(1) The Intervention Group (IG)

Subjects in the treatment group will be prescribed an individually EBCR program at home and in the hospital. The heart rate (HR) and walking distance targets individualized based on baseline 6MWT; and the intensity progression weekly if tolerated. Exercise education and exercise guidance book will be provided before the program begins. During the exercise in the hospital, subjects do a 45-minute exercise session consisting of a warm-up (5 minutes), core

HBECTR (Home-based Exercise Cardiac Telerehabilitation) study

exercise (30 minutes) in the form of walking on a treadmill, and cool down (10 minutes). This exercise is carried out twice a week over four weeks in the hospital (a total of 8 sessions) and is directly supervised by a certified cardiac rehab nurse (Table 2).

In this study, we will use web-based application (dashboard) to support the implementation of home-based exercise cardiac Telerehabilitation (HBECTR).

During exercise at home, subjects will be monitored by a telemonitoring system namely "*Telerehabilitasi Kardiovaskular*" or TEKAD. Patients wear a smart watch (CovWatch) that will be connected to a web-based system (http://tekad.covwatch.net). Before starting the exercise at home, the researcher and the IT team will ensure that the CovWatch is connected to the dashboard (App Rehab Cardio) to ensure the real-time monitoring of patient's vital sign during exercise (see figure 2). Home exercise is carried out for 45 minutes consisting of a 5-minute warm-up (e.g. simple stretching or slow walking), 30 minutes of core exercise walking based on the exercise dose and patient's ability, and 10 minutes of cooling down. Vital signs such as pulse, blood pressure, and oxygen saturation were monitored via smartwatch and displayed on the dashboard. Home exercises were performed 3 days a week over 4 weeks (12 sessions in total), with a schedule adjusted by the researchers so as not to coincide with the hospital exercise sessions. Subjects will be evaluated and receive weekly exercise reminders directly when they do exercise at hospital.

Real-time Monitoring

During exercise at home, subjects will be monitored by a nurse remotely trough a telemonitoring (TEKAD). With high-precision sensors, the smartwatch "covwatch" will continuously tracks the patient's heart rate, blood pressure, oxygen saturation level during exercise, and estimated walking distance. The system is connected via internet to the dashboard (App Rehab Cardio) (see figure 2).

Who provided:

Cardiac rehab Nurses, researcher, supervised by cardiologist

The Cardiologist will prescribe an exercise regime tailored by patients based on this baseline 6MWT, in accordance with the CR guidelines in Indonesia [21].

During exercise at the hospital, subjects from both groups will be supervised directly by CR nurses. Meanwhile, when the subjects of intervention group doing exercise at home, they will be monitored through a telemonitoring system using web-based application (dashboard) that has been developed by the researcher.

How (mode of delivery; individual or group):

The intervention group will receive hybrid or Combination of face-to-face hospital sessions and exercise at home supported with web-App for telemonitoring.

(1) The Intervention Group (IG)

Subjects in the treatment group will be prescribed an individually EBCR program at home and in the hospital. The heart rate (HR) and walking distance targets individualized based on baseline 6MWT; and the intensity progression weekly if tolerated. Exercise education and exercise guidance book will be provided before the program begins. During the exercise in the hospital, subjects do a 45-minute exercise session consisting of a warm-up (5 minutes), core exercise (30 minutes) in the form of walking on a treadmill, and cool down (10 minutes). This exercise is carried out twice a week over four weeks in the hospital (a total of 8 sessions) and is directly supervised by a certified cardiac rehab nurse (Table 2).

During exercise at home, subjects will be monitored by a telemonitoring system namely "*Telerehabilitasi Kardiovaskular*" or TEKAD. Patients wear a smart watch (CovWatch) that will be connected to a web-based system (http://tekad.covwatch.net). Before starting the exercise at home, the researcher and the IT team will ensure that the CovWatch is connected to the dashboard (App Rehab Cardio) to ensure the real-time monitoring of patient's vital sign during exercise (see figure 2). Home exercise is carried out for 45 minutes consisting of a 5-minute warm-up (e.g. simple stretching or slow walking), 30 minutes of core exercise walking based on the exercise dose and patient's ability, and 10 minutes of cooling down. Vital signs such as pulse, blood pressure, and oxygen saturation were monitored via smartwatch and displayed on the dashboard. Home exercises were performed 3 days a week over 4 weeks (12 sessions in

total), with a schedule adjusted by the researchers so as not to coincide with the hospital exercise sessions. Subjects will be evaluated and receive weekly exercise reminders directly when they do exercise at hospital.

Real-time Monitoring

During exercise at home, subjects will be monitored by a nurse remotely trough a telemonitoring (TEKAD). With high-precision sensors, the smartwatch “covwatch” will continuously tracks the patient’s heart rate, blood pressure, oxygen saturation level during exercise, and estimated walking distance. The system is connected via internet to the dashboard (App Rehab Cardio) (see figure 2).

(2) The Control Group (CG)

The control group will do a cardiac rehabilitation program in the hospital. Subjects in this group also received exercise education before starting the program. Subject will be scheduled 2 sessions per week or 8 sessions in total to do individual exercise at the hospital under CR nurse supervision. Based on hospital policy, patients are also suggested to do exercise at home independently (without monitoring). Hospital exercises included a 5-minute warm-up, 30-minute core exercise (walking on a treadmill), and a 10-minute cool-down (Table 2).

Where:

Hospital (supervised sessions) and patients’ homes (home sessions)

Settings

This study was being conducted since August 2024 and June 2025 at Dr. Sardjito Yogyakarta General Hospital, Indonesia, focusing on patients after percutaneous coronary intervention (PCI). The intervention will be done in CR center at Dr. Sardjito General Hospital, Yogyakarta and at patient’s home. The research environment included outpatient clinics and remote monitoring settings where participants received either the HBECTR Program or standard care. The setting ensured access to medical records, availability of telehealth technologies, and close coordination with healthcare professionals to support the implementation and evaluation of the HBECTR program.

When and how much:

5 sessions/week for 4 weeks; each session 45 minutes; 40–60% HRR

(1) The Intervention Group (IG)

Subjects in the treatment group will be prescribed an individually EBCR program at home and in the hospital. The heart rate (HR) and walking distance targets individualized based on baseline 6MWT; and the intensity progression weekly if tolerated. Exercise education and exercise guidance book will be provided before the program begins. During the exercise in the hospital, subjects do a 45-minute exercise session consisting of a warm-up (5 minutes), core exercise (30 minutes) in the form of walking on a treadmill, and cool down (10 minutes). This exercise is carried out twice a week over four weeks in the hospital (a total of 8 sessions) and is directly supervised by a certified cardiac rehab nurse (Table 2).

During exercise at home, subjects will be monitored by a telemonitoring system namely “*Telerehabilitasi Kardiovaskular*” or TEKAD. Patients wear a smart watch (CovWatch) that will be connected to a web-based system (http://tekad.covwatch.net). Before starting the exercise at home, the researcher and the IT team will ensure that the CovWatch is connected to the dashboard (App Rehab Cardio) to ensure the real-time monitoring of patient’s vital sign during exercise (see figure 2). Home exercise is carried out for 45 minutes consisting of a 5-minute warm-up (e.g. simple stretching or slow walking), 30 minutes of core exercise walking based on the exercise dose and patient’s ability, and 10 minutes of cooling down. Vital signs such as pulse, blood pressure, and oxygen saturation were monitored via smartwatch and displayed on the dashboard. Home exercises were performed 3 days a week over 4 weeks (12 sessions in total), with a schedule adjusted by the researchers so as not to coincide with the hospital exercise sessions. Subjects will be evaluated and receive weekly exercise reminders directly when they do exercise at hospital.

Real-time Monitoring

During exercise at home, subjects will be monitored by a nurse remotely trough a telemonitoring (TEKAD). With high-precision sensors, the smartwatch “covwatch” will continuously tracks the patient’s heart rate, blood pressure, oxygen saturation level during exercise, and estimated walking distance. The system is connected via internet to the dashboard (App Rehab Cardio) (see figure 2).

HBECTR (Home-based Exercise Cardiac Telerehabilitation) study

In this study, we will use web-based application (dashboard) to support the implementation of home-based exercise cardiac Telerehabilitation (HBECTR).

Table 2. Exercise prescription based on frequency, intensity, time, and type (FITT) for Control Group and Intervention Group

Tailoring:

Subjects in the treatment group will be prescribed an individually EBCR program at home and in the hospital. The heart rate (HR) and walking distance targets individualized based on baseline 6MWT; and the intensity progression weekly if tolerated.

How well (planned):

Measurement

Adherence to the exercise-based cardiac rehabilitation program as the primary outcome will be measured by recording the number of sessions attended by each participant. Based on previous study [42], adherence was calculated as the number of attended sessions divided by the total number of prescribed sessions, expressed as a percentage, with minimum 80% session completion considered adherent. Attendance during in-hospital exercise sessions was directly monitored by CR nurses and recorded in the CR attendance form, while home-based exercise attendance for IG was verified through the web-based dashboard system. Adherence monitored by smartwatch logs and dashboard analytics. This comprehensive approach ensured accurate measurement of each participant's adherence to the prescribed rehabilitation program.
