# Supplementary material for: Home-Based Exercise Cardiac Telerehabilitation on Adherence and Functional Capacity for Patients After Percutaneous Coronary Intervention in Indonesia: Protocol for a Quasi-Experimental Study
Source: JMIR Res Protoc. 2026 Jan 16;15:e81067. doi: 10.2196/81067 (PMC12810742; doi:10.2196/81067)
Supplement: Checklist 3 [file resprot-v15-e81067-s003.pdf]

CONSORT-EHEALTH (V 1.6.1) - Submission/Publication Form

The CONSORT-EHEALTH checklist is intended for authors of randomized trials evaluating web-based and Internet-based applications/interventions, including mobile interventions, electronic games (incl multiplayer games), social media, certain telehealth applications, and other interactive and/or networked electronic applications. Some of the items (e.g. all subitems under item 5 - description of the intervention) may also be applicable for other study designs.

The goal of the CONSORT EHEALTH checklist and guideline is to be
a) a guide for reporting for authors of RCTs,
b) to form a basis for appraisal of an ehealth trial (in terms of validity)

CONSORT-EHEALTH items/subitems are MANDATORY reporting items for studies published in the Journal of Medical Internet Research and other journals / scientific societies endorsing the checklist.

Items numbered 1., 2., 3., 4a., 4b etc are original CONSORT or CONSORT-NPT (non-pharmacologic treatment) items.
Items with Roman numerals (i., ii, iii, iv etc.) are CONSORT-EHEALTH extensions/clarifications.

As the CONSORT-EHEALTH checklist is still considered in a formative stage, we would ask that you also RATE ON A SCALE OF 1-5 how important/useful you feel each item is FOR THE PURPOSE OF THE CHECKLIST and reporting guideline (optional).

Mandatory reporting items are marked with a red *.
In the textboxes, either copy & paste the relevant sections from your manuscript into this form - please include any quotes from your manuscript in QUOTATION MARKS,
or answer directly by providing additional information not in the manuscript, or elaborating on why the item was not relevant for this study.

YOUR ANSWERS WILL BE PUBLISHED AS A SUPPLEMENTARY FILE TO YOUR PUBLICATION IN JMIR AND ARE CONSIDERED PART OF YOUR PUBLICATION (IF ACCEPTED).
Please fill in these questions diligently. Information will not be copyedited, so please use proper spelling and grammar, use correct capitalization, and avoid abbreviations.

DO NOT FORGET TO SAVE AS PDF _AND_ CLICK THE SUBMIT BUTTON SO YOUR ANSWERS ARE IN OUR DATABASE !!!

Citation Suggestion (if you append the pdf as Appendix we suggest to cite this paper in the caption):
Eysenbach G, CONSORT-EHEALTH Group
CONSORT-EHEALTH: Improving and Standardizing Evaluation Reports of Web-based and Mobile Health Interventions
J Med Internet Res 2011;13(4):e126

URL: http://www.jmir.org/2011/4/e126/
doi: 10.2196/jmir.1923
PMID: 22209829

wantiyah@mail.ugm.ac.id Switch account

Not shared

Draft saved

* Indicates required question

Your name *

First Last

Wantiyah Wantiyah

Primary Affiliation (short), City, Country *

University of Toronto, Toronto, Canada

Universitas Gadjah Mada, Yogyakarta, Indones

Your e-mail address *

abc@gmail.com

wantiyah@mail.ugm.ac.id

Title of your manuscript *

Provide the (draft) title of your manuscript.

Home-Based Exercise Cardiac Telerehabilitation (HBECTR) on Adherence and Functional Capacity for Patients After Percutaneous Coronary Intervention in Indonesia: Protocol for a Quasi-Experimental Study

Name of your App/Software/Intervention *

If there is a short and a long/alternate name, write the short name first and add the long name in brackets.

TEKAD (Telerehabilitasi Kardiovaskular)/HBEC

Evaluated Version (if any)

e.g. "V1", "Release 2017-03-01", "Version 2.0.27913"

Your answer

Language(s) *

What language is the intervention/app in? If multiple languages are available, separate by comma (e.g. "English, French")

Bahasa Indonesia

URL of your Intervention Website or App

e.g. a direct link to the mobile app on app in appstore (itunes, Google Play), or URL of the website. If the intervention is a DVD or hardware, you can also link to an Amazon page.

http://tekad.covwatch.net

URL of an image/screenshot (optional)

Your answer

Accessibility *

Can an enduser access the intervention presently?

- ◯ access is free and open
- ◉ access only for special usergroups, not open
- ◯ access is open to everyone, but requires payment/subscription/in-app purchases
- ◯ app/intervention no longer accessible
- ◯ Other:

Primary Medical Indication/Disease/Condition *

e.g. "Stress", "Diabetes", or define the target group in brackets after the condition, e.g. "Autism (Parents of children with)", "Alzheimers (Informal Caregivers of)"

Percutaneous Coronary Intervention (Patients

Primary Outcomes measured in trial *

comma-separated list of primary outcomes reported in the trial

Adherence

Secondary/other outcomes

Are there any other outcomes the intervention is expected to affect?

yes, the secondary objective was to determine the effect of HBECTR on patients’ functional capacity.

Recommended "Dose" *

What do the instructions for users say on how often the app should be used?

- ○ Approximately Daily
- ○ Approximately Weekly
- ○ Approximately Monthly
- ○ Approximately Yearly
- ◉ "as needed"
- ○ Other:

Approx. Percentage of Users (starters) still using the app as recommended after 3 months *

- ◉ unknown / not evaluated
- ○ 0-10%
- ○ 11-20%
- ○ 21-30%
- ○ 31-40%
- ○ 41-50%
- ○ 51-60%
- ○ 61-70%
- ○ 71%-80%
- ○ 81-90%
- ○ 91-100%
- ○ Other:

Overall, was the app/intervention effective? *

- ( ) yes: all primary outcomes were significantly better in intervention group vs control
- ( ) partly: SOME primary outcomes were significantly better in intervention group vs control
- (•) no statistically significant difference between control and intervention
- ( ) potentially harmful: control was significantly better than intervention in one or more outcomes
- ( ) inconclusive: more research is needed
- ( ) Other:

Article Preparation Status/Stage *

At which stage in your article preparation are you currently (at the time you fill in this form)

- ( ) not submitted yet - in early draft status
- ( ) not submitted yet - in late draft status, just before submission
- ( ) submitted to a journal but not reviewed yet
- (•) submitted to a journal and after receiving initial reviewer comments
- ( ) submitted to a journal and accepted, but not published yet
- ( ) published
- ( ) Other:

Journal *

If you already know where you will submit this paper (or if it is already submitted), please provide the journal name (if it is not JMIR, provide the journal name under "other")

- ◯ not submitted yet / unclear where I will submit this
- ◯ Journal of Medical Internet Research (JMIR)
- ◯ JMIR mHealth and UHealth
- ◯ JMIR Serious Games
- ◯ JMIR Mental Health
- ◯ JMIR Public Health
- ◯ JMIR Formative Research
- ◉ Other JMIR sister journal
- ◯ Other:

Is this a full powered effectiveness trial or a pilot/feasibility trial? *

- ◉ Pilot/feasibility
- ◯ Fully powered

Manuscript tracking number *

If this is a JMIR submission, please provide the manuscript tracking number under "other" (The ms tracking number can be found in the submission acknowledgement email, or when you login as author in JMIR. If the paper is already published in JMIR, then the ms tracking number is the four-digit number at the end of the DOI, to be found at the bottom of each published article in JMIR)

- ◯ no ms number (yet) / not (yet) submitted to / published in JMIR
- ◉ Other: 81067

TITLE AND ABSTRACT

1a) TITLE: Identification as a randomized trial in the title

1a) Does your paper address CONSORT item 1a? *

I.e does the title contain the phrase "Randomized Controlled Trial"? (if not, explain the reason under "other")

- ○ yes
- ◉ Other: This study is a Quasi-experimental study of HBECTR (web-based appl

1a-i) Identify the mode of delivery in the title

Identify the mode of delivery. Preferably use "web-based" and/or "mobile" and/or "electronic game" in the title. Avoid ambiguous terms like "online", "virtual", "interactive". Use "Internet-based" only if Intervention includes non-web-based Internet components (e.g. email), use "computer-based" or "electronic" only if offline products are used. Use "virtual" only in the context of "virtual reality" (3-D worlds). Use "online" only in the context of "online support groups". Complement or substitute product names with broader terms for the class of products (such as "mobile" or "smart phone" instead of "iphone"), especially if the application runs on different platforms.

	1	2	3	4	5	
subitem not at all important	○	○	○	○	○	essential

Does your paper address subitem 1a-i? *

Copy and paste relevant sections from manuscript title (include quotes in quotation marks "like this" to indicate direct quotes from your manuscript), or elaborate on this item by providing additional information not in the ms, or briefly explain why the item is not applicable/relevant for your study

Tittle: Home-Based Exercise Cardiac Telerehabilitation (HBECTR) on Adherence and Functional Capacity for Patients After Percutaneous Coronary Intervention in Indonesia: Protocol for a Quasi-Experimental Study

1a-ii) Non-web-based components or important co-interventions in title

Mention non-web-based components or important co-interventions in title, if any (e.g., “with telephone support”).

	1	2	3	4	5	
subitem not at all important	○	○	○	○	○	essential

Does your paper address subitem 1a-ii?

Copy and paste relevant sections from manuscript title (include quotes in quotation marks "like this" to indicate direct quotes from your manuscript), or elaborate on this item by providing additional information not in the ms, or briefly explain why the item is not applicable/relevant for your study

Your answer

1a-iii) Primary condition or target group in the title

Mention primary condition or target group in the title, if any (e.g., “for children with Type I Diabetes”) Example: A Web-based and Mobile Intervention with Telephone Support for Children with Type I Diabetes: Randomized Controlled Trial

	1	2	3	4	5	
subitem not at all important	○	○	○	◉	○	essential

Clear selection

Does your paper address subitem 1a-iii? *

Copy and paste relevant sections from manuscript title (include quotes in quotation marks "like this" to indicate direct quotes from your manuscript), or elaborate on this item by providing additional information not in the ms, or briefly explain why the item is not applicable/relevant for your study

yes
Home-Based Exercise Cardiac Telerehabilitation (HBECTR) on Adherence and Functional Capacity for Patients After Percutaneous Coronary Intervention in Indonesia: Protocol for a Quasi-Experimental Study

1b) ABSTRACT: Structured summary of trial design, methods, results, and conclusions

NPT extension: Description of experimental treatment, comparator, care providers, centers, and blinding status.

1b-i) Key features/functionalities/components of the intervention and comparator in the METHODS section of the ABSTRACT

Mention key features/functionalities/components of the intervention and comparator in the abstract. If possible, also mention theories and principles used for designing the site. Keep in mind the needs of systematic reviewers and indexers by including important synonyms. (Note: Only report in the abstract what the main paper is reporting. If this information is missing from the main body of text, consider adding it)

	1	2	3	4	5	
subitem not at all important	○	○	○	○	○	essential

Does your paper address subitem 1b-i? *

Copy and paste relevant sections from the manuscript abstract (include quotes in quotation marks "like this" to indicate direct quotes from your manuscript), or elaborate on this item by providing additional information not in the ms, or briefly explain why the item is not applicable/relevant for your study

ABSTRACT

Background: Home-based cardiac rehabilitation (HBCR) is a promising approach to improve access and adherence to cardiac rehabilitation (CR), which remains underutilized. However, monitoring patient adherence and safety during home-based exercise is challenging. Therefore, develop and implement a home-based exercise cardiac telerehabilitation (HBECTR) program, integrating structured exercise guidance with remote monitoring.
Objectives: This trial is designed as a pilot feasibility study to evaluate the practicality, safety, and preliminary effects of HBECTR in patients who have undergone percutaneous coronary intervention (PCI). The primary objective is to analyse the effect of HBECTR on patients' adherence to CR, while the secondary objective is to measure the effect of HBECTR on the functional capacity of post-PCI patients.
Methods: This study is a quasi-experimental non-equivalent control group pretest–post test. The eligible participants are adults (≥18 years), CAD patients after successful PCI, and has active health insurance. Subjects will be consecutively recruited from Dr. Sardjito General Hospital, Yogyakarta, Indonesia with minimum 60 post-PCI patients as samples, divided into two groups an intervention and comparison/control group. The intervention group will receive hybrid cardiac rehabilitation. Patients will be scheduled to do exercise twice a week at hospital, and three times a week at patients' home. When patients exercise at home, they have to wear the smart watch "CovWatch", that will be connected to the web-based application (App Rehab Cardio) to monitor their vital signs and the distance of walking. While, the control group will get usual care (CBCR). Data will be analyzed using student t-test if the data comply the normality assumption. This protocol received approval from institutional review board (IRB) of Faculty of Medicine, Public Health, dan Nursing UGM (approval number: KE/FK/0850/EC/2024).
Results: The recruitment of the subject started from August 2024. This study is being funded since February 2025. During the submission, we have recruited 63 subjects for both intervention and control group.
Conclusions: It is expected that HBECTR program can be feasible and safely implemented in LMICs, particularly in Indonesia. By integrating a telerehabilitation model, it is expected that HBECTR will improve patients' adherence in participate to CR, and also increase the functional capacity of patients after PCI.

1b-ii) Level of human involvement in the METHODS section of the ABSTRACT

Clarify the level of human involvement in the abstract, e.g., use phrases like “fully automated” vs. “therapist/nurse/care provider/physician-assisted” (mention number and expertise of providers involved, if any). (Note: Only report in the abstract what the main paper is reporting. If this information is missing from the main body of text, consider adding it)

	1	2	3	4	5	
subitem not at all important	○	○	○	○	○	essential

Does your paper address subitem 1b-ii?

Copy and paste relevant sections from the manuscript abstract (include quotes in quotation marks "like this" to indicate direct quotes from your manuscript), or elaborate on this item by providing additional information not in the ms, or briefly explain why the item is not applicable/relevant for your study

Your answer

1b-iii) Open vs. closed, web-based (self-assessment) vs. face-to-face assessments in the METHODS section of the ABSTRACT

Mention how participants were recruited (online vs. offline), e.g., from an open access website or from a clinic or a closed online user group (closed usergroup trial), and clarify if this was a purely web-based trial, or there were face-to-face components (as part of the intervention or for assessment). Clearly say if outcomes were self-assessed through questionnaires (as common in web-based trials). Note: In traditional offline trials, an open trial (open-label trial) is a type of clinical trial in which both the researchers and participants know which treatment is being administered. To avoid confusion, use “blinded” or “unblinded” to indicated the level of blinding instead of “open”, as “open” in web-based trials usually refers to “open access” (i.e. participants can self-enrol). (Note: Only report in the abstract what the main paper is reporting. If this information is missing from the main body of text, consider adding it)

	1	2	3	4	5	
subitem not at all important	○	○	○	○	○	essential

Does your paper address subitem 1b-iii?

Copy and paste relevant sections from the manuscript abstract (include quotes in quotation marks "like this" to indicate direct quotes from your manuscript), or elaborate on this item by providing additional information not in the ms, or briefly explain why the item is not applicable/relevant for your study

Your answer

1b-iv) RESULTS section in abstract must contain use data

Report number of participants enrolled/assessed in each group, the use/uptake of the intervention (e.g., attrition/adherence metrics, use over time, number of logins etc.), in addition to primary/secondary outcomes. (Note: Only report in the abstract what the main paper is reporting. If this information is missing from the main body of text, consider adding it)

	1	2	3	4	5	
subitem not at all important	○	○	○	○	○	essential

Does your paper address subitem 1b-iv?

Copy and paste relevant sections from the manuscript abstract (include quotes in quotation marks "like this" to indicate direct quotes from your manuscript), or elaborate on this item by providing additional information not in the ms, or briefly explain why the item is not applicable/relevant for your study

Your answer

1b-v) CONCLUSIONS/DISCUSSION in abstract for negative trials

Conclusions/Discussions in abstract for negative trials: Discuss the primary outcome - if the trial is negative (primary outcome not changed), and the intervention was not used, discuss whether negative results are attributable to lack of uptake and discuss reasons. (Note: Only report in the abstract what the main paper is reporting. If this information is missing from the main body of text, consider adding it)

	1	2	3	4	5	
subitem not at all important	○	○	○	○	○	essential

Does your paper address subitem 1b-v?

Copy and paste relevant sections from the manuscript abstract (include quotes in quotation marks "like this" to indicate direct quotes from your manuscript), or elaborate on this item by providing additional information not in the ms, or briefly explain why the item is not applicable/relevant for your study

Your answer

INTRODUCTION

2a) In INTRODUCTION: Scientific background and explanation of rationale

2a-i) Problem and the type of system/solution

Describe the problem and the type of system/solution that is object of the study: intended as stand-alone intervention vs. incorporated in broader health care program? Intended for a particular patient population? Goals of the intervention, e.g., being more cost-effective to other interventions, replace or complement other solutions? (Note: Details about the intervention are provided in “Methods” under 5)

	1	2	3	4	5	
subitem not at all important	○	○	○	○	○	essential

Does your paper address subitem 2a-i? *

Copy and paste relevant sections from the manuscript (include quotes in quotation marks "like this" to indicate direct quotes from your manuscript), or elaborate on this item by providing additional information not in the ms, or briefly explain why the item is not applicable/relevant for your study

Despite the various advantages and benefits of HBCR, there are several obstacles, such as the absence of clear guidelines regarding dosage and components, its implementation is still limited to certain cases, and it has not been implemented in all countries. Research and implementation of HBCR are mostly in developed countries such as America, Australia, and England [5], [25].
Adding artificial intelligence that is able to interpret data and monitor the implementation of CR can increase the safety and feasibility of HBCR. Devices installed or wearied on patients are expected to identify the patients' toleration of exercise dose and monitoring the vital signs to ensure patient safety during exercise at home [22]. The use of information technology in its implementation is predicted to increase the long-term effects of HBCR [25]. Smartphones or mobile devices (20/31, 65%), web-based portals (18/31, 58%), and email-SMS (11/31, 35%) are the most often used of information technology to support CR [14]. In this study, we will use web-based application (dashboard) to support the implementation of home-based exercise cardiac Telerehabilitation (HBECTR).
Although many support the implementation of telerehabilitation, there is still no definitive data on the efficacy of HBECTR compared to CBCR, so it still requires further research [26]. Besides, the biggest challenge today is how to improve patient adherence and compliance in CR, the development of HBCR guidelines, and the implementation of information technology in HBCR that can be applied in countries with limited resources, like Indonesia. Therefore, this study is designed as a pilot feasibility trial to evaluate the practicality, safety, and short-term effects of a digitally supported home-based exercise cardiac telerehabilitation (HBECTR) program in Indonesian post-PCI patients. This is the first pilot study in Indonesia implementing digitally supported HBCR, particularly using web-based platform, addressing a critical gap in LMIC settings where CR utilization remains low. The pilot nature of this study allows us to examine the feasibility of recruitment, adherence, safety monitoring, and digital health implementation in a resource-limited setting.

2a-ii) Scientific background, rationale: What is known about the (type of) system

Scientific background, rationale: What is known about the (type of) system that is the object of the study (be sure to discuss the use of similar systems for other conditions/diagnoses, if appropiate), motivation for the study, i.e. what are the reasons for and what is the context for this specific study, from which stakeholder viewpoint is the study performed, potential impact of findings [2]. Briefly justify the choice of the comparator.

	1	2	3	4	5	
subitem not at all important	○	○	○	○	○	essential

Does your paper address subitem 2a-ii? *

Copy and paste relevant sections from the manuscript (include quotes in quotation marks "like this" to indicate direct quotes from your manuscript), or elaborate on this item by providing additional information not in the ms, or briefly explain why the item is not applicable/relevant for your study

Although many support the implementation of telerehabilitation, there is still no definitive data on the efficacy of HBECTR compared to CBCR, so it still requires further research [26]. Besides, the biggest challenge today is how to improve patient adherence and compliance in CR, the development of HBCR guidelines, and the implementation of information technology in HBCR that can be applied in countries with limited resources, like Indonesia. Therefore, this study is designed as a pilot feasibility trial to evaluate the practicality, safety, and short-term effects of a digitally supported home-based exercise cardiac telerehabilitation (HBECTR) program in Indonesian post-PCI patients. This is the first pilot study in Indonesia implementing digitally supported HBCR, particularly using web-based platform, addressing a critical gap in LMIC settings where CR utilization remains low. The pilot nature of this study allows us to examine the feasibility of recruitment, adherence, safety monitoring, and digital health implementation in a resource-limited setting.

2b) In INTRODUCTION: Specific objectives or hypotheses

Does your paper address CONSORT subitem 2b? *

Copy and paste relevant sections from the manuscript (include quotes in quotation marks "like this" to indicate direct quotes from your manuscript), or elaborate on this item by providing additional information not in the ms, or briefly explain why the item is not applicable/relevant for your study

OBJECTIVES
This study aimed to evaluate the effectiveness of a home-based exercise cardiac telerehabilitation (HBECTR) program for patients after percutaneous coronary intervention (PCI). The primary objective was to assess the effect of HBECTR on adherence to cardiac rehabilitation (CR), while the secondary objective was to determine its effect on patients' functional capacity.

Hypotheses:
1. There is no difference of functional capacity between HBECTR group and control group (H0) or HBECTR will improve functional capacity compared to control (H1)
2. There is no difference of adherence between HBECTR group and control group (H0) or HBECTR will improve adherence to CR compared to control (H1)

METHODS

3a) Description of trial design (such as parallel, factorial) including allocation ratio

Does your paper address CONSORT subitem 3a? *

Copy and paste relevant sections from the manuscript (include quotes in quotation marks "like this" to indicate direct quotes from your manuscript), or elaborate on this item by providing additional information not in the ms, or briefly explain why the item is not applicable/relevant for your study

Study Design
This trial is designed as a pilot feasibility study to evaluate the practicality, safety, and preliminary effects of home-based exercise cardiac telerehabilitation (HBECTR) using a quasi-experimental non-equivalent control group pretest–post-test. Participants are assigned to either the intervention group, which receives the HBECTR program, or the control group, which receives standard care (CBCR). Pre- and post-intervention data will be collected to assess the impact of the HBECTR program on patients' adherence as primary outcome and their functional capacity as secondary outcome.
We use a quasi-experimental design with a non-randomized control group due to the limited number of eligible participants available during the recruitment, the logistical and resource constraints, and to bringing the implementation closer to the real world. As a pilot study, we want to identify the feasibility of HBECTR in Indonesia. In Fact, there is a strong need to further investigate and incorporate synchronous digital CR into the routine of cardiac patients' secondary prevention routine [29].

3b) Important changes to methods after trial commencement (such as eligibility criteria), with reasons

Does your paper address CONSORT subitem 3b? *

Copy and paste relevant sections from the manuscript (include quotes in quotation marks "like this" to indicate direct quotes from your manuscript), or elaborate on this item by providing additional information not in the ms, or briefly explain why the item is not applicable/relevant for your study

yes, because of the limitation number of subjects, we amendment the eligibility criteria. We recruit all patients after PCI, not limited to the patients after primary PCI.

3b-i) Bug fixes, Downtimes, Content Changes

Bug fixes, Downtimes, Content Changes: ehealth systems are often dynamic systems. A description of changes to methods therefore also includes important changes made on the intervention or comparator during the trial (e.g., major bug fixes or changes in the functionality or content) (5-iii) and other “unexpected events” that may have influenced study design such as staff changes, system failures/downtimes, etc. [2].

	1	2	3	4	5	
subitem not at all important	○	○	○	○	○	essential

Does your paper address subitem 3b-i?

Copy and paste relevant sections from the manuscript (include quotes in quotation marks "like this" to indicate direct quotes from your manuscript), or elaborate on this item by providing additional information not in the ms, or briefly explain why the item is not applicable/relevant for your study

Your answer

4a) Eligibility criteria for participants

Does your paper address CONSORT subitem 4a? *

Copy and paste relevant sections from the manuscript (include quotes in quotation marks "like this" to indicate direct quotes from your manuscript), or elaborate on this item by providing additional information not in the ms, or briefly explain why the item is not applicable/relevant for your study

Samples
We will carefully select the samples based on the inclusion criteria were: (1) CAD patient after successful PCI, (2) aged over 18 years, (3) covered by active health insurance (BPJS), (4) willing to participate and sign informed consent, (5) having internet connectivity at home (for the intervention group), (6) living with family or having a family member as a caregiver, and (7) able to read and understand Bahasa Indonesia.

4a-i) Computer / Internet literacy

Computer / Internet literacy is often an implicit “de facto” eligibility criterion - this should be explicitly clarified.

	1	2	3	4	5	
subitem not at all important	○	○	○	○	○	essential

Does your paper address subitem 4a-i?

Copy and paste relevant sections from the manuscript (include quotes in quotation marks "like this" to indicate direct quotes from your manuscript), or elaborate on this item by providing additional information not in the ms, or briefly explain why the item is not applicable/relevant for your study

Your answer

4a-ii) Open vs. closed, web-based vs. face-to-face assessments:

Open vs. closed, web-based vs. face-to-face assessments: Mention how participants were recruited (online vs. offline), e.g., from an open access website or from a clinic, and clarify if this was a purely web-based trial, or there were face-to-face components (as part of the intervention or for assessment), i.e., to what degree got the study team to know the participant. In online-only trials, clarify if participants were quasi-anonymous and whether having multiple identities was possible or whether technical or logistical measures (e.g., cookies, email confirmation, phone calls) were used to detect/prevent these.

	1	2	3	4	5	
subitem not at all important	○	○	○	○	○	essential

Does your paper address subitem 4a-ii? *

Copy and paste relevant sections from the manuscript (include quotes in quotation marks "like this" to indicate direct quotes from your manuscript), or elaborate on this item by providing additional information not in the ms, or briefly explain why the item is not applicable/relevant for your study

The control group will receive standard hospital or center-based CR (CBCR), while the treatment group will receive Home-based exercise cardiac telerehabilitation (HBECTR). We define HBECTR as an exercise-based cardiac rehabilitation conducted in a hybrid setting, both in the hospital and at the patient's home, supported by a telemonitoring system. During exercise at the hospital, subjects from both groups will be supervised directly by CR nurses. Meanwhile, when the subjects of intervention group doing exercise at home, they will be monitored through a telemonitoring system using web-based application (dashboard) that has been developed by the researcher. The researcher team developed and conducted usability testing before the dashboard released (reported separately). During exercise at home, the subject will wear a smart watch (Covwatch) produced by PT Covwatch Karya Nusantara (PT CKN) Indonesia, which record the patient's vital signs and estimated walking distance. The system is connected via internet to the dashboard at the hospital (App Rehab Cardio) "Telerehabilitasi Kardiovaskular" or TEKAD. Patients wear a smart watch (CovWatch) that will be connected to a web-based system (http://tekad.covwatch.net). Before starting the exercise at home, the researcher and the IT team will ensure that the CovWatch is connected to the dashboard (App Rehab Cardio) to ensure the real-time monitoring of patient's vital sign during exercise (see figure 2).

4a-iii) Information giving during recruitment

Information given during recruitment. Specify how participants were briefed for recruitment and in the informed consent procedures (e.g., publish the informed consent documentation as appendix, see also item X26), as this information may have an effect on user self-selection, user expectation and may also bias results.

	1	2	3	4	5	
subitem not at all important	○	○	○	○	○	essential

Does your paper address subitem 4a-iii?

Copy and paste relevant sections from the manuscript (include quotes in quotation marks "like this" to indicate direct quotes from your manuscript), or elaborate on this item by providing additional information not in the ms, or briefly explain why the item is not applicable/relevant for your study

Your answer

4b) Settings and locations where the data were collected

Does your paper address CONSORT subitem 4b? *

Copy and paste relevant sections from the manuscript (include quotes in quotation marks "like this" to indicate direct quotes from your manuscript), or elaborate on this item by providing additional information not in the ms, or briefly explain why the item is not applicable/relevant for your study

Settings
This study was being conducted since August 2024 and June 2025 at Dr. Sardjito Yogyakarta General Hospital, Indonesia, focusing on patients after percutaneous coronary intervention (PCI). The intervention will be done in CR center at Dr. Sardjito General Hospital, Yogyakarta and at patient's home. The research environment included outpatient clinics and remote monitoring settings where participants received either the HBECTR Program or standard care. The setting ensured access to medical records, availability of telehealth technologies, and close coordination with healthcare professionals to support the implementation and evaluation of the HBECTR program.

4b-i) Report if outcomes were (self-)assessed through online questionnaires

Clearly report if outcomes were (self-)assessed through online questionnaires (as common in web-based trials) or otherwise.

	1	2	3	4	5	
subitem not at all important	○	○	○	○	○	essential

Does your paper address subitem 4b-i? *

Copy and paste relevant sections from the manuscript (include quotes in quotation marks "like this" to indicate direct quotes from your manuscript), or elaborate on this item by providing additional information not in the ms, or briefly explain why the item is not applicable/relevant for your study

Measurement

Adherence to the exercise-based cardiac rehabilitation program as the primary outcome will be measured by recording the number of sessions attended by each participant. Based on previous study [42], adherence was calculated as the number of attended sessions divided by the total number of prescribed sessions, expressed as a percentage, with minimum 80% session completion considered adherent. Attendance during in-hospital exercise sessions was directly monitored by CR nurses and recorded in the CR attendance form, while home-based exercise attendance for IG was verified through the web-based dashboard system. Adherence monitored by smartwatch logs and dashboard analytics. This comprehensive approach ensured accurate measurement of each participant's adherence to the prescribed rehabilitation program.
Functional capacity will be assessed using the Six-Minute Walk Test (6MWT) to measure the total distance (in meters) during six minutes test (six-minute walk distance/6MWD) [21], [37]. In addition, physiological data such as heart rate, blood pressure, and oxygen saturation will be continuously monitored using telemonitoring system (see figure 2), ensuring safety and accuracy of HBECTR. To provide a more comprehensive assessment of functional improvement, peak oxygen uptake (VO_2 peak) or metabolic equivalents (METs) were estimated based on the distance covered in the 6MWT, using established predictive equations [43], [44]. These measurements collectively provided robust data on the participants' functional performance following the intervention.

4b-ii) Report how institutional affiliations are displayed

Report how institutional affiliations are displayed to potential participants [on ehealth media], as affiliations with prestigious hospitals or universities may affect volunteer rates, use, and reactions with regards to an intervention.(Not a required item – describe only if this may bias results)

	1	2	3	4	5	
subitem not at all important	○	○	○	○	○	essential

Does your paper address subitem 4b-ii?

Copy and paste relevant sections from the manuscript (include quotes in quotation marks "like this" to indicate direct quotes from your manuscript), or elaborate on this item by providing additional information not in the ms, or briefly explain why the item is not applicable/relevant for your study

Your answer

5) The interventions for each group with sufficient details to allow replication, including how and when they were actually administered

5-i) Mention names, credential, affiliations of the developers, sponsors, and owners

Mention names, credential, affiliations of the developers, sponsors, and owners [6] (if authors/evaluators are owners or developer of the software, this needs to be declared in a “Conflict of interest” section or mentioned elsewhere in the manuscript).

	1	2	3	4	5	
subitem not at all important	○	○	○	○	○	essential

Does your paper address subitem 5-i?

Copy and paste relevant sections from the manuscript (include quotes in quotation marks "like this" to indicate direct quotes from your manuscript), or elaborate on this item by providing additional information not in the ms, or briefly explain why the item is not applicable/relevant for your study

Your answer

5-ii) Describe the history/development process

Describe the history/development process of the application and previous formative evaluations (e.g., focus groups, usability testing), as these will have an impact on adoption/use rates and help with interpreting results.

	1	2	3	4	5	
subitem not at all important	○	○	○	○	○	essential

Does your paper address subitem 5-ii?

Copy and paste relevant sections from the manuscript (include quotes in quotation marks "like this" to indicate direct quotes from your manuscript), or elaborate on this item by providing additional information not in the ms, or briefly explain why the item is not applicable/relevant for your study

Your answer

5-iii) Revisions and updating

Revisions and updating. Clearly mention the date and/or version number of the application/intervention (and comparator, if applicable) evaluated, or describe whether the intervention underwent major changes during the evaluation process, or whether the development and/or content was “frozen” during the trial. Describe dynamic components such as news feeds or changing content which may have an impact on the replicability of the intervention (for unexpected events see item 3b).

	1	2	3	4	5	
subitem not at all important	○	○	○	○	○	essential

Does your paper address subitem 5-iii?

Copy and paste relevant sections from the manuscript (include quotes in quotation marks "like this" to indicate direct quotes from your manuscript), or elaborate on this item by providing additional information not in the ms, or briefly explain why the item is not applicable/relevant for your study

Your answer

5-iv) Quality assurance methods

Provide information on quality assurance methods to ensure accuracy and quality of information provided [1], if applicable.

	1	2	3	4	5	
subitem not at all important	○	○	○	○	○	essential

Does your paper address subitem 5-iv?

Copy and paste relevant sections from the manuscript (include quotes in quotation marks "like this" to indicate direct quotes from your manuscript), or elaborate on this item by providing additional information not in the ms, or briefly explain why the item is not applicable/relevant for your study

Your answer

5-v) Ensure replicability by publishing the source code, and/or providing screenshots/screen-capture video, and/or providing flowcharts of the algorithms used

Ensure replicability by publishing the source code, and/or providing screenshots/screen-capture video, and/or providing flowcharts of the algorithms used. Replicability (i.e., other researchers should in principle be able to replicate the study) is a hallmark of scientific reporting.

	1	2	3	4	5	
subitem not at all important	○	○	○	○	○	essential

Does your paper address subitem 5-v?

Copy and paste relevant sections from the manuscript (include quotes in quotation marks "like this" to indicate direct quotes from your manuscript), or elaborate on this item by providing additional information not in the ms, or briefly explain why the item is not applicable/relevant for your study

Your answer

5-vi) Digital preservation

Digital preservation: Provide the URL of the application, but as the intervention is likely to change or disappear over the course of the years; also make sure the intervention is archived (Internet Archive, webcitation.org, and/or publishing the source code or screenshots/videos alongside the article). As pages behind login screens cannot be archived, consider creating demo pages which are accessible without login.

	1	2	3	4	5	
subitem not at all important	○	○	○	○	○	essential

Does your paper address subitem 5-vi?

Copy and paste relevant sections from the manuscript (include quotes in quotation marks "like this" to indicate direct quotes from your manuscript), or elaborate on this item by providing additional information not in the ms, or briefly explain why the item is not applicable/relevant for your study

Your answer

5-vii) Access

Access: Describe how participants accessed the application, in what setting/context, if they had to pay (or were paid) or not, whether they had to be a member of specific group. If known, describe how participants obtained “access to the platform and Internet” [1]. To ensure access for editors/reviewers/readers, consider to provide a “backdoor” login account or demo mode for reviewers/readers to explore the application (also important for archiving purposes, see vi).

	1	2	3	4	5	
subitem not at all important	○	○	○	○	○	essential

Does your paper address subitem 5-vii? *

Copy and paste relevant sections from the manuscript (include quotes in quotation marks "like this" to indicate direct quotes from your manuscript), or elaborate on this item by providing additional information not in the ms, or briefly explain why the item is not applicable/relevant for your study

(1) The Intervention Group (IG)
Subjects in the treatment group will be prescribed an individually EBCR program at home and in the hospital. The heart rate (HR) and walking distance targets individualized based on baseline 6MWT; and the intensity progression weekly if tolerated. Exercise education and exercise guidance book will be provided before the program begins. During the exercise in the hospital, subjects do a 45-minute exercise session consisting of a warm-up (5 minutes), core exercise (30 minutes) in the form of walking on a treadmill, and cool down (10 minutes). This exercise is carried out twice a week over four weeks in the hospital (a total of 8 sessions) and is directly supervised by a certified cardiac rehab nurse (Table 2).
During exercise at home, subjects will be monitored by a telemonitoring system namely "Telerehabilitasi Kardiovaskular" or TEKAD. Patients wear a smart watch (CovWatch) that will be connected to a web-based system (http://tekad.covwatch.net). Before starting the exercise at home, the researcher and the IT team will ensure that the CovWatch is connected to the dashboard (App Rehab Cardio) to ensure the real-time monitoring of patient's vital sign during exercise (see figure 2). Home exercise is carried out for 45 minutes consisting of a 5-minute warm-up (e.g. simple stretching or slow walking), 30 minutes of core exercise walking based on the exercise dose and patient's ability, and 10 minutes of cooling down. Vital signs such as pulse, blood pressure, and oxygen saturation were monitored via smartwatch and displayed on the dashboard. Home exercises were performed 3 days a week over 4 weeks (12 sessions in total), with a schedule adjusted by the researchers so as not to coincide with the hospital exercise sessions. Subjects will be evaluated and receive weekly exercise reminders directly when they do exercise at hospital.
Real-time Monitoring
During exercise at home, subjects will be monitored by a nurse remotely trough a telemonitoring (TEKAD). With high-precision sensors, the smartwatch "covwatch" will continuously tracks the patient's heart rate, blood pressure, oxygen saturation level during exercise, and estimated walking distance. The system is connected via internet to the dashboard (App Rehab Cardio) (see figure 2).

5-viii) Mode of delivery, features/functionalities/components of the intervention and comparator, and the theoretical framework

Describe mode of delivery, features/functionalities/components of the intervention and comparator, and the theoretical framework [6] used to design them (instructional strategy [1], behaviour change techniques, persuasive features, etc., see e.g., [7, 8] for terminology). This includes an in-depth description of the content (including where it is coming from and who developed it) [1]," whether [and how] it is tailored to individual circumstances and allows users to track their progress and receive feedback" [6]. This also includes a description of communication delivery channels and – if computer-mediated communication is a component – whether communication was synchronous or asynchronous [6]. It also includes information on presentation strategies [1], including page design principles, average amount of text on pages, presence of hyperlinks to other resources, etc. [1].

	1	2	3	4	5	
subitem not at all important	○	○	○	○	○	essential

Does your paper address subitem 5-viii? *

Copy and paste relevant sections from the manuscript (include quotes in quotation marks "like this" to indicate direct quotes from your manuscript), or elaborate on this item by providing additional information not in the ms, or briefly explain why the item is not applicable/relevant for your study

(1) The Intervention Group (IG)
Subjects in the treatment group will be prescribed an individually EBCR program at home and in the hospital. The heart rate (HR) and walking distance targets individualized based on baseline 6MWT; and the intensity progression weekly if tolerated. Exercise education and exercise guidance book will be provided before the program begins. During the exercise in the hospital, subjects do a 45-minute exercise session consisting of a warm-up (5 minutes), core exercise (30 minutes) in the form of walking on a treadmill, and cool down (10 minutes). This exercise is carried out twice a week over four weeks in the hospital (a total of 8 sessions) and is directly supervised by a certified cardiac rehab nurse (Table 2).
During exercise at home, subjects will be monitored by a telemonitoring system namely “Telerehabilitasi Kardiovaskular” or TEKAD. Patients wear a smart watch (CovWatch) that will be connected to a web-based system (http://tekad.covwatch.net). Before starting the exercise at home, the researcher and the IT team will ensure that the CovWatch is connected to the dashboard (App Rehab Cardio) to ensure the real-time monitoring of patient’s vital sign during exercise (see figure 2). Home exercise is carried out for 45 minutes consisting of a 5-minute warm-up (e.g. simple stretching or slow walking), 30 minutes of core exercise walking based on the exercise dose and patient’s ability, and 10 minutes of cooling down. Vital signs such as pulse, blood pressure, and oxygen saturation were monitored via smartwatch and displayed on the dashboard. Home exercises were performed 3 days a week over 4 weeks (12 sessions in total), with a schedule adjusted by the researchers so as not to coincide with the hospital exercise sessions. Subjects will be evaluated and receive weekly exercise reminders directly when they do exercise at hospital.
Real-time Monitoring
During exercise at home, subjects will be monitored by a nurse remotely trough a telemonitoring (TEKAD). With high-precision sensors, the smartwatch “covwatch” will continuously tracks the patient’s heart rate, blood pressure, oxygen saturation level during exercise, and estimated walking distance. The system is connected via internet to the dashboard (App Rehab Cardio) (see figure 2).

(2) The Control Group (CG)
The control group will do a cardiac rehabilitation program in the hospital. Subjects in this group also received exercise education before starting the program. Subject will be scheduled 2 sessions per week or 8 sessions in total to do individual exercise at the hospital under CR nurse supervision. Based on hospital policy, patients are also suggested to do exercise at home independently (without monitoring). Hospital exercises included a 5-minute warm-up, 30-minute core exercise (walking on a treadmill), and a 10-minute cool-down (Table 2).

5-ix) Describe use parameters

Describe use parameters (e.g., intended “doses” and optimal timing for use). Clarify what instructions or recommendations were given to the user, e.g., regarding timing, frequency, heaviness of use, if any, or was the intervention used ad libitum.

	1	2	3	4	5	
subitem not at all important	○	○	○	○	○	essential

Does your paper address subitem 5-ix?

Copy and paste relevant sections from the manuscript (include quotes in quotation marks "like this" to indicate direct quotes from your manuscript), or elaborate on this item by providing additional information not in the ms, or briefly explain why the item is not applicable/relevant for your study

Your answer

5-x) Clarify the level of human involvement

Clarify the level of human involvement (care providers or health professionals, also technical assistance) in the e-intervention or as co-intervention (detail number and expertise of professionals involved, if any, as well as “type of assistance offered, the timing and frequency of the support, how it is initiated, and the medium by which the assistance is delivered”. It may be necessary to distinguish between the level of human involvement required for the trial, and the level of human involvement required for a routine application outside of a RCT setting (discuss under item 21 – generalizability).

	1	2	3	4	5	
subitem not at all important	○	○	○	○	○	essential

Does your paper address subitem 5-x?

Copy and paste relevant sections from the manuscript (include quotes in quotation marks "like this" to indicate direct quotes from your manuscript), or elaborate on this item by providing additional information not in the ms, or briefly explain why the item is not applicable/relevant for your study

Your answer

5-xi) Report any prompts/reminders used

Report any prompts/reminders used: Clarify if there were prompts (letters, emails, phone calls, SMS) to use the application, what triggered them, frequency etc. It may be necessary to distinguish between the level of prompts/reminders required for the trial, and the level of prompts/reminders for a routine application outside of a RCT setting (discuss under item 21 – generalizability).

	1	2	3	4	5	
subitem not at all important	○	○	○	○	○	essential

Does your paper address subitem 5-xi? *

Copy and paste relevant sections from the manuscript (include quotes in quotation marks "like this" to indicate direct quotes from your manuscript), or elaborate on this item by providing additional information not in the ms, or briefly explain why the item is not applicable/relevant for your study

Subjects will be evaluated and receive weekly exercise reminders directly when they do exercise at hospital.

5-xii) Describe any co-interventions (incl. training/support)

Describe any co-interventions (incl. training/support): Clearly state any interventions that are provided in addition to the targeted eHealth intervention, as ehealth intervention may not be designed as stand-alone intervention. This includes training sessions and support [1]. It may be necessary to distinguish between the level of training required for the trial, and the level of training for a routine application outside of a RCT setting (discuss under item 21 – generalizability.

	1	2	3	4	5	
subitem not at all important	○	○	○	○	○	essential

Does your paper address subitem 5-xii? *

Copy and paste relevant sections from the manuscript (include quotes in quotation marks "like this" to indicate direct quotes from your manuscript), or elaborate on this item by providing additional information not in the ms, or briefly explain why the item is not applicable/relevant for your study

Exercise education and exercise guidance book will be provided before the program begins.

6a) Completely defined pre-specified primary and secondary outcome measures, including how and when they were assessed

Does your paper address CONSORT subitem 6a? *

Copy and paste relevant sections from the manuscript (include quotes in quotation marks "like this" to indicate direct quotes from your manuscript), or elaborate on this item by providing additional information not in the ms, or briefly explain why the item is not applicable/relevant for your study

Adherence to the exercise-based cardiac rehabilitation program as the primary outcome will be measured by recording the number of sessions attended by each participant. Based on previous study [42], adherence was calculated as the number of attended sessions divided by the total number of prescribed sessions, expressed as a percentage, with minimum 80% session completion considered adherent. Attendance during in-hospital exercise sessions was directly monitored by CR nurses and recorded in the CR attendance form, while home-based exercise attendance for IG was verified through the web-based dashboard system. Adherence monitored by smartwatch logs and dashboard analytics. This comprehensive approach ensured accurate measurement of each participant's adherence to the prescribed rehabilitation program.
Functional capacity will be assessed using the Six-Minute Walk Test (6MWT) to measure the total distance (in meters) during six minutes test (six-minute walk distance/6MWD) [21], [37].
The primary and secondary outcome will be measured after participants finished the intervention (4 weeks follow up).

6a-i) Online questionnaires: describe if they were validated for online use and apply CHERRIES items to describe how the questionnaires were designed/deployed

If outcomes were obtained through online questionnaires, describe if they were validated for online use and apply CHERRIES items to describe how the questionnaires were designed/deployed [9].

	1	2	3	4	5	
subitem not at all important	○	○	○	○	○	essential

Does your paper address subitem 6a-i?

Copy and paste relevant sections from manuscript text

Your answer

6a-ii) Describe whether and how “use” (including intensity of use/dosage) was defined/measured/monitored

Describe whether and how “use” (including intensity of use/dosage) was defined/measured/monitored (logins, logfile analysis, etc.). Use/adoption metrics are important process outcomes that should be reported in any ehealth trial.

	1	2	3	4	5	
subitem not at all important	○	○	○	○	○	essential

Does your paper address subitem 6a-ii?

Copy and paste relevant sections from manuscript text

Your answer

6a-iii) Describe whether, how, and when qualitative feedback from participants was obtained

Describe whether, how, and when qualitative feedback from participants was obtained (e.g., through emails, feedback forms, interviews, focus groups).

	1	2	3	4	5	
subitem not at all important	○	○	○	○	○	essential

Does your paper address subitem 6a-iii?

Copy and paste relevant sections from manuscript text

Your answer

6b) Any changes to trial outcomes after the trial commenced, with reasons

Does your paper address CONSORT subitem 6b? *

Copy and paste relevant sections from the manuscript (include quotes in quotation marks "like this" to indicate direct quotes from your manuscript), or elaborate on this item by providing additional information not in the ms, or briefly explain why the item is not applicable/relevant for your study

None planned at registration; deviations will be reported

7a) How sample size was determined

NPT: When applicable, details of whether and how the clustering by care provides or centers was addressed

7a-i) Describe whether and how expected attrition was taken into account when calculating the sample size

Describe whether and how expected attrition was taken into account when calculating the sample size.

	1	2	3	4	5	
subitem not at all important	○	○	○	○	○	essential

Does your paper address subitem 7a-i?

Copy and paste relevant sections from manuscript title (include quotes in quotation marks "like this" to indicate direct quotes from your manuscript), or elaborate on this item by providing additional information not in the ms, or briefly explain why the item is not applicable/relevant for your study

Your answer

7b) When applicable, explanation of any interim analyses and stopping guidelines

Does your paper address CONSORT subitem 7b? *

Copy and paste relevant sections from the manuscript (include quotes in quotation marks "like this" to indicate direct quotes from your manuscript), or elaborate on this item by providing additional information not in the ms, or briefly explain why the item is not applicable/relevant for your study

None planned. This study will examine the feasibility of the HBECTR program, adherence, and safety monitoring

8a) Method used to generate the random allocation sequence

NPT: When applicable, how care providers were allocated to each trial group

Does your paper address CONSORT subitem 8a? *

Copy and paste relevant sections from the manuscript (include quotes in quotation marks "like this" to indicate direct quotes from your manuscript), or elaborate on this item by providing additional information not in the ms, or briefly explain why the item is not applicable/relevant for your study

Not randomized; quasi-experimental, consecutive sampling

We use a quasi-experimental design with a non-randomized control group. Both groups will be recruited consecutively using identical eligibility criteria, recruitment procedures, and measurement protocols. The control/comparison group was recruited and completed follow-up before initiation of the intervention group.

8b) Type of randomisation; details of any restriction (such as blocking and block size)

Does your paper address CONSORT subitem 8b? *

Copy and paste relevant sections from the manuscript (include quotes in quotation marks "like this" to indicate direct quotes from your manuscript), or elaborate on this item by providing additional information not in the ms, or briefly explain why the item is not applicable/relevant for your study

We use a quasi-experimental design with a non-randomized control group. Both groups will be recruited consecutively using identical eligibility criteria, recruitment procedures, and measurement protocols.

9) Mechanism used to implement the random allocation sequence (such as sequentially numbered containers), describing any steps taken to conceal the sequence until interventions were assigned

Does your paper address CONSORT subitem 9? *

Copy and paste relevant sections from the manuscript (include quotes in quotation marks "like this" to indicate direct quotes from your manuscript), or elaborate on this item by providing additional information not in the ms, or briefly explain why the item is not applicable/relevant for your study

We use a quasi-experimental design with a non-randomized control group. Both groups will be recruited consecutively using identical eligibility criteria, recruitment procedures, and measurement protocols. The control/comparison group was recruited and completed follow-up before initiation of the intervention group.

10) Who generated the random allocation sequence, who enrolled participants, and who assigned participants to interventions

Does your paper address CONSORT subitem 10? *

Copy and paste relevant sections from the manuscript (include quotes in quotation marks "like this" to indicate direct quotes from your manuscript), or elaborate on this item by providing additional information not in the ms, or briefly explain why the item is not applicable/relevant for your study

We use a quasi-experimental design with a non-randomized control group. Both groups will be recruited consecutively using identical eligibility criteria, recruitment procedures, and measurement protocols. The control/comparison group was recruited and completed follow-up before initiation of the intervention group.

11a) If done, who was blinded after assignment to interventions (for example, participants, care providers, those assessing outcomes) and how

NPT: Whether or not administering co-interventions were blinded to group assignment

11a-i) Specify who was blinded, and who wasn't

Specify who was blinded, and who wasn't. Usually, in web-based trials it is not possible to blind the participants [1, 3] (this should be clearly acknowledged), but it may be possible to blind outcome assessors, those doing data analysis or those administering co-interventions (if any).

	1	2	3	4	5	
subitem not at all important	○	○	○	○	○	essential

Does your paper address subitem 11a-i? *

Copy and paste relevant sections from the manuscript (include quotes in quotation marks "like this" to indicate direct quotes from your manuscript), or elaborate on this item by providing additional information not in the ms, or briefly explain why the item is not applicable/relevant for your study

Blinding procedures are often not feasible in quasi-experimental studies due to the absence of randomized assignment and the practical realities of real-world intervention delivery, particularly in exercise, physical activity, and rehabilitation research [36]. In this study, full blinding of participants and assessors was not possible because of the overt nature of the intervention and logistical constraints. Nevertheless, the risk of bias was mitigated by implementing several strategies: using validated measurement tools; providing objective and standardized outcome assessments; and, wherever feasible, ensuring that data analysts were blinded to group allocation during the data analysis phase. Data were coded such that the analysts were unaware of group assignments until after the completion of initial analysis.

11a-ii) Discuss e.g., whether participants knew which intervention was the “intervention of interest” and which one was the “comparator”

Informed consent procedures (4a-ii) can create biases and certain expectations - discuss e.g., whether participants knew which intervention was the “intervention of interest” and which one was the “comparator”.

	1	2	3	4	5	
subitem not at all important	○	○	○	○	○	essential

Does your paper address subitem 11a-ii?

Copy and paste relevant sections from the manuscript (include quotes in quotation marks "like this" to indicate direct quotes from your manuscript), or elaborate on this item by providing additional information not in the ms, or briefly explain why the item is not applicable/relevant for your study

Your answer

11b) If relevant, description of the similarity of interventions
(this item is usually not relevant for ehealth trials as it refers to similarity of a placebo or sham intervention to a active medication/intervention)

Does your paper address CONSORT subitem 11b? *

Copy and paste relevant sections from the manuscript (include quotes in quotation marks "like this" to indicate direct quotes from your manuscript), or elaborate on this item by providing additional information not in the ms, or briefly explain why the item is not applicable/relevant for your study

Intervention group: HBECTR
Control/comparison: usual care (CBCR)

12a) Statistical methods used to compare groups for primary and secondary outcomes
NPT: When applicable, details of whether and how the clustering by care providers or centers was addressed

Does your paper address CONSORT subitem 12a? *

Copy and paste relevant sections from the manuscript (include quotes in quotation marks "like this" to indicate direct quotes from your manuscript), or elaborate on this item by providing additional information not in the ms, or briefly explain why the item is not applicable/relevant for your study

Statistical Analysis Planning
Descriptive statistics will summarize feasibility outcomes (recruitment, adherence, completion rates, safety events), while inferential tests (paired t-tests or ANCOVA) will be used to explore within- and between-group changes (including means and SDs) in functional capacity and adherence measures. Statistical significance will be interpreted cautiously, given the pilot nature and limited sample size of the study [33], [36].

To analyzing the effects of the HBECTR on patient adherence as primary outcome and functional capacity as secondary outcome there is no formal multiplicity adjustment planned. The data analysis based on normality assumption tested using the Shapiro-Wilk test. If the data is complying the normal assumption, we will use student t-test, and vice versa, non-parametric tests will be used if assumptions are violated. In all statistical tests, a P<.05 (95 % CI) was considered statistically significant.

12a-i) Imputation techniques to deal with attrition / missing values

Imputation techniques to deal with attrition / missing values: Not all participants will use the intervention/comparator as intended and attrition is typically high in ehealth trials. Specify how participants who did not use the application or dropped out from the trial were treated in the statistical analysis (a complete case analysis is strongly discouraged, and simple imputation techniques such as LOCF may also be problematic [4]).

	1	2	3	4	5	
subitem not at all important	○	○	○	○	○	essential

Does your paper address subitem 12a-i? *

Copy and paste relevant sections from the manuscript (include quotes in quotation marks "like this" to indicate direct quotes from your manuscript), or elaborate on this item by providing additional information not in the ms, or briefly explain why the item is not applicable/relevant for your study

We will use multiple imputation for missing data. Multiple imputation (MI) is a widely used technique for handling missing data in various fields, including medical, social, and ecological research.

12b) Methods for additional analyses, such as subgroup analyses and adjusted analyses

Does your paper address CONSORT subitem 12b? *

Copy and paste relevant sections from the manuscript (include quotes in quotation marks "like this" to indicate direct quotes from your manuscript), or elaborate on this item by providing additional information not in the ms, or briefly explain why the item is not applicable/relevant for your study

baseline demographic and clinical characteristics will be carefully documented, and differences between groups will be statistically adjusted using multivariable regression

X26) REB/IRB Approval and Ethical Considerations [recommended as subheading under "Methods"] (not a CONSORT item)

X26-i) Comment on ethics committee approval

	1	2	3	4	5	
subitem not at all important	○	○	○	○	○	essential

Does your paper address subitem X26-i?

Copy and paste relevant sections from the manuscript (include quotes in quotation marks "like this" to indicate direct quotes from your manuscript), or elaborate on this item by providing additional information not in the ms, or briefly explain why the item is not applicable/relevant for your study

Your answer

x26-ii) Outline informed consent procedures

Outline informed consent procedures e.g., if consent was obtained offline or online (how? Checkbox, etc.?), and what information was provided (see 4a-ii). See [6] for some items to be included in informed consent documents.

	1	2	3	4	5	
subitem not at all important	○	○	○	○	○	essential

Does your paper address subitem X26-ii?

Copy and paste relevant sections from the manuscript (include quotes in quotation marks "like this" to indicate direct quotes from your manuscript), or elaborate on this item by providing additional information not in the ms, or briefly explain why the item is not applicable/relevant for your study

Your answer

X26-iii) Safety and security procedures

Safety and security procedures, incl. privacy considerations, and any steps taken to reduce the likelihood or detection of harm (e.g., education and training, availability of a hotline)

	1	2	3	4	5	
subitem not at all important	○	○	○	○	○	essential

Does your paper address subitem X26-iii?

Copy and paste relevant sections from the manuscript (include quotes in quotation marks "like this" to indicate direct quotes from your manuscript), or elaborate on this item by providing additional information not in the ms, or briefly explain why the item is not applicable/relevant for your study

Your answer

RESULTS

13a) For each group, the numbers of participants who were randomly assigned, received intended treatment, and were analysed for the primary outcome

NPT: The number of care providers or centers performing the intervention in each group and the number of patients treated by each care provider in each center

Does your paper address CONSORT subitem 13a? *

Copy and paste relevant sections from the manuscript (include quotes in quotation marks "like this" to indicate direct quotes from your manuscript), or elaborate on this item by providing additional information not in the ms, or briefly explain why the item is not applicable/relevant for your study

RESULTS
The recruitment of the subject started from August 2024. This study is being funded since February 2025. During the submission, we have recruited 63 subjects for both intervention and control group. The intervention for both groups finished at June 2025 and the data is being analyzed.

13b) For each group, losses and exclusions after randomisation, together with reasons

Does your paper address CONSORT subitem 13b? (NOTE: Preferably, this is shown in a CONSORT flow diagram) *

Copy and paste relevant sections from the manuscript (include quotes in quotation marks "like this" to indicate direct quotes from your manuscript), or elaborate on this item by providing additional information not in the ms, or briefly explain why the item is not applicable/relevant for your study

CONSORT-style flow diagram included (figure 1)

13b-i) Attrition diagram

Strongly recommended: An attrition diagram (e.g., proportion of participants still logging in or using the intervention/comparator in each group plotted over time, similar to a survival curve) or other figures or tables demonstrating usage/dose/engagement.

	1	2	3	4	5	
subitem not at all important	○	○	○	○	○	essential

Does your paper address subitem 13b-i?

Copy and paste relevant sections from the manuscript or cite the figure number if applicable (include quotes in quotation marks "like this" to indicate direct quotes from your manuscript), or elaborate on this item by providing additional information not in the ms, or briefly explain why the item is not applicable/relevant for your study

Your answer

14a) Dates defining the periods of recruitment and follow-up

Does your paper address CONSORT subitem 14a? *

Copy and paste relevant sections from the manuscript (include quotes in quotation marks "like this" to indicate direct quotes from your manuscript), or elaborate on this item by providing additional information not in the ms, or briefly explain why the item is not applicable/relevant for your study

RESULTS
The recruitment of the subject started from August 2024. This study is being funded since February 2025. During the submission, we have recruited 63 subjects for both intervention and control group. The intervention for both groups finished at June 2025 and the data is being analyzed.

14a-i) Indicate if critical “secular events” fell into the study period

Indicate if critical “secular events” fell into the study period, e.g., significant changes in Internet resources available or “changes in computer hardware or Internet delivery resources”

	1	2	3	4	5	
subitem not at all important	○	○	○	○	○	essential

Does your paper address subitem 14a-i?

Copy and paste relevant sections from the manuscript (include quotes in quotation marks "like this" to indicate direct quotes from your manuscript), or elaborate on this item by providing additional information not in the ms, or briefly explain why the item is not applicable/relevant for your study

Your answer

14b) Why the trial ended or was stopped (early)

Does your paper address CONSORT subitem 14b? *

Copy and paste relevant sections from the manuscript (include quotes in quotation marks "like this" to indicate direct quotes from your manuscript), or elaborate on this item by providing additional information not in the ms, or briefly explain why the item is not applicable/relevant for your study

RESULTS
The recruitment of the subject started from August 2024. This study is being funded since February 2025. During the submission, we have recruited 63 subjects for both intervention and control group. The intervention for both groups finished in June 2025 and the data is being analyzed.

15) A table showing baseline demographic and clinical characteristics for each group

NPT: When applicable, a description of care providers (case volume, qualification, expertise, etc.) and centers (volume) in each group

Does your paper address CONSORT subitem 15? *

Copy and paste relevant sections from the manuscript (include quotes in quotation marks "like this" to indicate direct quotes from your manuscript), or elaborate on this item by providing additional information not in the ms, or briefly explain why the item is not applicable/relevant for your study

not yet reported (NA) in this protocol. The data is being analyzed.

15-i) Report demographics associated with digital divide issues

In ehealth trials it is particularly important to report demographics associated with digital divide issues, such as age, education, gender, social-economic status, computer/Internet/ehealth literacy of the participants, if known.

	1	2	3	4	5	
subitem not at all important	○	○	○	○	○	essential

Does your paper address subitem 15-i? *

Copy and paste relevant sections from the manuscript (include quotes in quotation marks "like this" to indicate direct quotes from your manuscript), or elaborate on this item by providing additional information not in the ms, or briefly explain why the item is not applicable/relevant for your study

not yet reported (NA) in this protocol. The data is being analyzed. It will be reported in another article.

Measurement
In this study, respondents will be asked to fill out the participant characteristics, including age, sex, body mass index (BMI), and medical history, which were collected at baseline through direct interviews and medical record reviews. Sociodemographic information, such as marital status, educational level, occupation, insurance ownership, and average monthly income, was also gathered. In addition, participants were asked about their smoking history (categorized as current, former, or never smoked) and any past or current illnesses, diseases, or risk factors. Clinical information was obtained, including the patient's medical diagnosis, ejection fraction (EF), prescribed therapies, weight and height, as well as the schedule of heart polyclinic control and cardiac rehabilitation, if applicable.

16) For each group, number of participants (denominator) included in each analysis and whether the analysis was by original assigned groups

16-i) Report multiple “denominators” and provide definitions

Report multiple “denominators” and provide definitions: Report N’s (and effect sizes) “across a range of study participation [and use] thresholds” [1], e.g., N exposed, N consented, N used more than x times, N used more than y weeks, N participants “used” the intervention/comparator at specific pre-defined time points of interest (in absolute and relative numbers per group). Always clearly define “use” of the intervention.

	1	2	3	4	5	
subitem not at all important	○	○	○	○	○	essential

Does your paper address subitem 16-i? *

Copy and paste relevant sections from the manuscript (include quotes in quotation marks "like this" to indicate direct quotes from your manuscript), or elaborate on this item by providing additional information not in the ms, or briefly explain why the item is not applicable/relevant for your study

RESULTS
The recruitment of the subject started from August 2024. This study is being funded since February 2025. During the submission, we have recruited 63 subjects for both intervention and control group. The intervention for both groups finished in June 2025 and the data is being analyzed.

16-ii) Primary analysis should be intent-to-treat

Primary analysis should be intent-to-treat, secondary analyses could include comparing only “users”, with the appropriate caveats that this is no longer a randomized sample (see 18-i).

	1	2	3	4	5	
subitem not at all important	○	○	○	○	○	essential

Does your paper address subitem 16-ii?

Copy and paste relevant sections from the manuscript (include quotes in quotation marks "like this" to indicate direct quotes from your manuscript), or elaborate on this item by providing additional information not in the ms, or briefly explain why the item is not applicable/relevant for your study

Your answer

17a) For each primary and secondary outcome, results for each group, and the estimated effect size and its precision (such as 95% confidence interval)

Does your paper address CONSORT subitem 17a? *

Copy and paste relevant sections from the manuscript (include quotes in quotation marks "like this" to indicate direct quotes from your manuscript), or elaborate on this item by providing additional information not in the ms, or briefly explain why the item is not applicable/relevant for your study

To analyzing the effects of the HBECTR on patient adherence as primary outcome and functional capacity as secondary outcome there is no formal multiplicity adjustment planned. The data analysis based on normality assumption tested using the Shapiro-Wilk test. If the data is complying the normal assumption, we will use student t-test, and vice versa, non-parametric tests will be used if assumptions are violated. In all statistical tests, a P<.05 (95 % CI) was considered statistically significant.

17a-i) Presentation of process outcomes such as metrics of use and intensity of use

In addition to primary/secondary (clinical) outcomes, the presentation of process outcomes such as metrics of use and intensity of use (dose, exposure) and their operational definitions is critical. This does not only refer to metrics of attrition (13-b) (often a binary variable), but also to more continuous exposure metrics such as “average session length”. These must be accompanied by a technical description how a metric like a “session” is defined (e.g., timeout after idle time) [1] (report under item 6a).

	1	2	3	4	5	
subitem not at all important	○	○	○	○	○	essential

Does your paper address subitem 17a-i?

Copy and paste relevant sections from the manuscript (include quotes in quotation marks "like this" to indicate direct quotes from your manuscript), or elaborate on this item by providing additional information not in the ms, or briefly explain why the item is not applicable/relevant for your study

Your answer

17b) For binary outcomes, presentation of both absolute and relative effect sizes is recommended

Does your paper address CONSORT subitem 17b? *

Copy and paste relevant sections from the manuscript (include quotes in quotation marks "like this" to indicate direct quotes from your manuscript), or elaborate on this item by providing additional information not in the ms, or briefly explain why the item is not applicable/relevant for your study

Yes, adherence was calculated as the number of attended sessions divided by the total number of prescribed sessions, expressed as a percentage, with minimum 80% session completion considered adherent.

18) Results of any other analyses performed, including subgroup analyses and adjusted analyses, distinguishing pre-specified from exploratory

Does your paper address CONSORT subitem 18? *

Copy and paste relevant sections from the manuscript (include quotes in quotation marks "like this" to indicate direct quotes from your manuscript), or elaborate on this item by providing additional information not in the ms, or briefly explain why the item is not applicable/relevant for your study

In addition, propensity score adjustment will be applied in exploratory models to control for potential confounding variables (e.g., age, sex, baseline functional capacity, comorbidity

18-i) Subgroup analysis of comparing only users

A subgroup analysis of comparing only users is not uncommon in ehealth trials, but if done, it must be stressed that this is a self-selected sample and no longer an unbiased sample from a randomized trial (see 16-iii).

	1	2	3	4	5	
subitem not at all important	○	○	○	○	○	essential

Does your paper address subitem 18-i?

Copy and paste relevant sections from the manuscript (include quotes in quotation marks "like this" to indicate direct quotes from your manuscript), or elaborate on this item by providing additional information not in the ms, or briefly explain why the item is not applicable/relevant for your study

Your answer

19) All important harms or unintended effects in each group

(for specific guidance see CONSORT for harms)

Does your paper address CONSORT subitem 19? *

Copy and paste relevant sections from the manuscript (include quotes in quotation marks "like this" to indicate direct quotes from your manuscript), or elaborate on this item by providing additional information not in the ms, or briefly explain why the item is not applicable/relevant for your study

Safety and Monitoring

Participants will receive detailed instructions on the prescribed exercise regimen to be performed both at the hospital and at home. They will be guided on the appropriate type, intensity, and duration of exercise, and will be encouraged to report any symptoms or discomfort experienced during exercise. If patients experience any adverse symptoms—such as chest pain, shortness of breath, unusual fatigue, or other discomfort—they will be instructed to immediately stop exercising and rest.

During exercise at home, subjects will be able to contact the cardiac rehabilitation team via WhatsApp in case of any complaints or concerns. Conversely, if the team detects abnormal changes in vital signs (e.g., heart rate, blood pressure, or oxygen saturation) during remote monitoring, they will proactively contact the patient to inquire about their condition. Then, if necessary, patients will be advised to stop exercise and take adequate rest.

Furthermore, the risk of AE and SAEs in EBCR is low. However, it is increased during vigorous intensity exercise relative to rest [49], and intensive exercise training (≥7 x/week) [50]. Therefore, this study will implement the moderate intensity exercise within 5 times per week. Besides, the exercise prescription will be individualized based on the 6MWT baseline, following the CR guideline in Indonesia [21]. All adverse events (AE) or serious adverse events (SAE) during the study will be reported to the Cardiologist who is supervising this program. These also will be documented case report form (CRF) and reported to the ethical board.

19-i) Include privacy breaches, technical problems

Include privacy breaches, technical problems. This does not only include physical "harm" to participants, but also incidents such as perceived or real privacy breaches [1], technical problems, and other unexpected/unintended incidents. "Unintended effects" also includes unintended positive effects [2].

	1	2	3	4	5	
subitem not at all important	○	○	○	○	○	essential

Does your paper address subitem 19-i?

Copy and paste relevant sections from the manuscript (include quotes in quotation marks "like this" to indicate direct quotes from your manuscript), or elaborate on this item by providing additional information not in the ms, or briefly explain why the item is not applicable/relevant for your study

Your answer

19-ii) Include qualitative feedback from participants or observations from staff/researchers

Include qualitative feedback from participants or observations from staff/researchers, if available, on strengths and shortcomings of the application, especially if they point to unintended/unexpected effects or uses. This includes (if available) reasons for why people did or did not use the application as intended by the developers.

	1	2	3	4	5	
subitem not at all important	○	○	○	○	○	essential

Does your paper address subitem 19-ii?

Copy and paste relevant sections from the manuscript (include quotes in quotation marks "like this" to indicate direct quotes from your manuscript), or elaborate on this item by providing additional information not in the ms, or briefly explain why the item is not applicable/relevant for your study

Your answer

DISCUSSION

22) Interpretation consistent with results, balancing benefits and harms, and considering other relevant evidence

NPT: In addition, take into account the choice of the comparator, lack of or partial blinding, and unequal expertise of care providers or centers in each group

22-i) Restate study questions and summarize the answers suggested by the data, starting with primary outcomes and process outcomes (use)

Restate study questions and summarize the answers suggested by the data, starting with primary outcomes and process outcomes (use).

	1	2	3	4	5	
subitem not at all important	○	○	○	○	○	essential

Does your paper address subitem 22-i? *

Copy and paste relevant sections from the manuscript (include quotes in quotation marks "like this" to indicate direct quotes from your manuscript), or elaborate on this item by providing additional information not in the ms, or briefly explain why the item is not applicable/relevant for your study

DISCUSSION
The low adherence of patients to participate in CR programs, particularly after PCI remains a significant challenge in Indonesia and other countries. Despite class 1 recommendations for CR as part of secondary prevention, CBCR are often underutilized due to various factors, including transportation difficulties, limited availability, and low patient motivation. This study aims to address these challenges by implementing a HBECTR program to enhance patients' adherence to CR.

22-ii) Highlight unanswered new questions, suggest future research

Highlight unanswered new questions, suggest future research.

	1	2	3	4	5	
subitem not at all important	○	○	○	○	○	essential

Does your paper address subitem 22-ii?

Copy and paste relevant sections from the manuscript (include quotes in quotation marks "like this" to indicate direct quotes from your manuscript), or elaborate on this item by providing additional information not in the ms, or briefly explain why the item is not applicable/relevant for your study

Your answer

20) Trial limitations, addressing sources of potential bias, imprecision, and, if relevant, multiplicity of analyses

20-i) Typical limitations in ehealth trials

Typical limitations in ehealth trials: Participants in ehealth trials are rarely blinded. Ehealth trials often look at a multiplicity of outcomes, increasing risk for a Type I error. Discuss biases due to non-use of the intervention/usability issues, biases through informed consent procedures, unexpected events.

	1	2	3	4	5	
subitem not at all important	○	○	○	○	○	essential

Does your paper address subitem 20-i? *

Copy and paste relevant sections from the manuscript (include quotes in quotation marks "like this" to indicate direct quotes from your manuscript), or elaborate on this item by providing additional information not in the ms, or briefly explain why the item is not applicable/relevant for your study

Furthermore, several potential limitations should be acknowledged in this quasi-experimental study. First, the intervention may yield null or minimal effects, particularly on adherence to cardiac rehabilitation and functional capacity, due to variability in participant engagement, baseline fitness levels, or external barriers such as comorbidities or limited motivation. Second, feasibility challenges are anticipated, including variable access to technology, differences in digital literacy, difficulties in remotely monitoring safety, and potential missing data from non-adherence or technical issues. Using wearable device (smart watch) is preferrable by most patients, however it has challenge in its implementation and need further exploration to gain deeper understanding on patients' needs and preference [52]. Finally, resource limitations may constrain implementation, including limited personnel for supervision, insufficient devices for remote monitoring, costs associated with web-based platforms, and the need for staff training and scheduling coordination.

21) Generalisability (external validity, applicability) of the trial findings

NPT: External validity of the trial findings according to the intervention, comparators, patients, and care providers or centers involved in the trial

21-i) Generalizability to other populations

Generalizability to other populations: In particular, discuss generalizability to a general Internet population, outside of a RCT setting, and general patient population, including applicability of the study results for other organizations

	1	2	3	4	5	
subitem not at all important	○	○	○	○	○	essential

Does your paper address subitem 21-i?

Copy and paste relevant sections from the manuscript (include quotes in quotation marks "like this" to indicate direct quotes from your manuscript), or elaborate on this item by providing additional information not in the ms, or briefly explain why the item is not applicable/relevant for your study

In addition, this study also has limitations including its modest sample size and short follow-up duration limits its generalizability to the broader population. These are intentional features of a pilot feasibility trial that focus on testing feasibility, safety, and preliminary efficacy signals rather than providing conclusive evidence of effectiveness [34].

21-ii) Discuss if there were elements in the RCT that would be different in a routine application setting

Discuss if there were elements in the RCT that would be different in a routine application setting (e.g., prompts/reminders, more human involvement, training sessions or other co-interventions) and what impact the omission of these elements could have on use, adoption, or outcomes if the intervention is applied outside of a RCT setting.

	1	2	3	4	5	
subitem not at all important	○	○	○	○	○	essential

Does your paper address subitem 21-ii?

Copy and paste relevant sections from the manuscript (include quotes in quotation marks "like this" to indicate direct quotes from your manuscript), or elaborate on this item by providing additional information not in the ms, or briefly explain why the item is not applicable/relevant for your study

Your answer

OTHER INFORMATION

23) Registration number and name of trial registry

Does your paper address CONSORT subitem 23? *

Copy and paste relevant sections from the manuscript (include quotes in quotation marks "like this" to indicate direct quotes from your manuscript), or elaborate on this item by providing additional information not in the ms, or briefly explain why the item is not applicable/relevant for your study

This protocol has been submitted to ANZCTR for registration: ANZCTR (390528); https://www.anzctr.org.au/Trial/MyTrial.aspx

24) Where the full trial protocol can be accessed, if available

Does your paper address CONSORT subitem 24? *

Cite a Multimedia Appendix, other reference, or copy and paste relevant sections from the manuscript (include quotes in quotation marks "like this" to indicate direct quotes from your manuscript), or elaborate on this item by providing additional information not in the ms, or briefly explain why the item is not applicable/relevant for your study

Wantiyah Wantiyah et al. Home-Based Exercise Cardiac Telerehabilitation (HBECTR) on Adherence and Functional Capacity for Patients After Percutaneous Coronary Intervention in Indonesia: Protocol for a Quasi-Experimental Study. JMIR Research Protocols (submitted). doi:10.2196/81067

25) Sources of funding and other support (such as supply of drugs), role of funders

Does your paper address CONSORT subitem 25? *

Copy and paste relevant sections from the manuscript (include quotes in quotation marks "like this" to indicate direct quotes from your manuscript), or elaborate on this item by providing additional information not in the ms, or briefly explain why the item is not applicable/relevant for your study

This study was supported by the DAMAS Grant from Faculty of Medicine, Public Health, and Nursing Universitas Gadjah Mada, Indonesia. The funder did not involve in the protocol development.

X27) Conflicts of Interest (not a CONSORT item)

X27-i) State the relation of the study team towards the system being evaluated

In addition to the usual declaration of interests (financial or otherwise), also state the relation of the study team towards the system being evaluated, i.e., state if the authors/evaluators are distinct from or identical with the developers/sponsors of the intervention.

	1	2	3	4	5	
subitem not at all important	○	○	○	○	○	essential

Does your paper address subitem X27-i?

Copy and paste relevant sections from the manuscript (include quotes in quotation marks "like this" to indicate direct quotes from your manuscript), or elaborate on this item by providing additional information not in the ms, or briefly explain why the item is not applicable/relevant for your study

Your answer

About the CONSORT EHEALTH checklist

As a result of using this checklist, did you make changes in your manuscript? *

- ◉ yes, major changes
- ○ yes, minor changes
- ○ no

What were the most important changes you made as a result of using this checklist?

Your answer

How much time did you spend on going through the checklist INCLUDING making changes in your manuscript *

we need for at least one month to revise the manuscript

As a result of using this checklist, do you think your manuscript has improved? *

- (●) yes
- ( ) no
- ( ) Other:

Would you like to become involved in the CONSORT EHEALTH group?

This would involve for example becoming involved in participating in a workshop and writing an "Explanation and Elaboration" document

- ( ) yes
- ( ) no
- ( ) Other:

Any other comments or questions on CONSORT EHEALTH

Your answer

STOP - Save this form as PDF before you click submit

To generate a record that you filled in this form, we recommend to generate a PDF of this page (on a Mac, simply select "print" and then select "print as PDF") before you submit it.

When you submit your (revised) paper to JMIR, please upload the PDF as supplementary file.

Don't worry if some text in the textboxes is cut off, as we still have the complete information in our database. Thank you!

Final step: Click submit !

Click submit so we have your answers in our database!

Submit Clear form

Never submit passwords through Google Forms.

This form was created outside of your domain. - Contact form owner - Terms of Service - Privacy Policy

Does this form look suspicious? Report

Google Forms
